# Progress and Prospects of Precursor Preparation for Practical Superconducting Wires and Tapes

**DOI:** 10.3390/ma19214759

**Published:** 2026-11-09

**Authors:** Xiaolong Zhu, He Huang, Shuhua Yang, Cong Liu, Chen Guo, Chiheng Dong, Xianping Zhang, Dongliang Wang, Yanwei Ma

**Affiliations:** 1 School of Material Science and Engineering, University of Jinan, Jinan 250022, China; 2 Beijing Key Laboratory of Applied Superconductivity, Institute of Electrical Engineering, Chinese Academy of Sciences, Beijing 100190, China; 3 Shandong Key Laboratory of Advanced Electromagnetic Conversion Technology, Institute of Electrical Engineering and Advanced Electromagnetic Drive Technology, Qilu Zhongke, Jinan 250013, China; 4 University of Chinese Academy of Sciences, Beijing 100049, China

**Keywords:** superconducting wires and tapes, powder-in-tube method, BSCCO, MgB_2_, IBS, precursor powders

## Abstract

Superconducting wires and tapes are key enabling materials for high-field magnets, nuclear fusion, superconducting power systems and medical imaging. In practical conductors fabricated by the powder-in-tube (PIT) method, the precursor powder is not merely a starting material; it establishes the chemical, microstructural and processing conditions that govern phase formation, texture, density, connectivity and ultimately the critical current density. The challenge is therefore to develop precursor powders that deliver high superconducting performance while remaining compatible with stable, cost-effective and scalable wire and tape manufacturing. This review examines the preparation of precursor powders for practical BSCCO, MgB_2_ and 122-type iron-based superconductors. We first clarify the functions and evaluation criteria of precursor powders, and then compare the principal synthesis routes and powder-modification strategies used for these conductor systems. Particular attention is given to the links between composition, impurity phases, particle characteristics, reactivity, heat-treatment windows and the resulting conductor properties. We further discuss how non-uniformity and process sensitivity of powders can hinder batch consistency and obstruct scale-up. Overall, the evidence shows that progress must go beyond particle-size refinement or reactivity enhancement alone. A more useful target is an engineering-oriented powder system with precise composition, controllable phase composition, designable particle structure, a matched heat-treatment window and robust production yield. Such powder design, coupled with conductor process optimization, provides a practical path toward higher-performance and more reliable superconducting wires and tapes.

## 1. Introduction

Large-scale applications of superconducting materials have long been constrained by two fundamental issues. The first is whether the intrinsic properties of the material can satisfy the temperature and magnetic field requirements, and the second is whether practical conductors can be fabricated at low cost with excellent batch-to-batch stability. Application-oriented superconducting conductors are not laboratory samples pursuing high critical temperature; rather, they are required to possess high critical current, satisfactory mechanical strength, low AC losses, superior thermal stability and mass production feasibility under complex service conditions. In recent years, numerous studies on practical superconducting materials have confirmed that manufacturing cost, machinability and length consistency remain the key factors restricting widespread application [1].

Superconducting materials that are currently available or hold practical promise mainly include the following categories: NbTi, Nb_3_Sn, magnesium diboride (MgB_2_), bismuth-based superconductors (BSCCO), rare-earth barium copper oxide (REBCO) superconductors and iron-based superconductors (IBS).

The fabrication routes for superconducting wires and tapes vary among different types of superconductors. Among them, NbTi, as a binary alloy, features a relatively simple fabrication route and can be produced via vacuum melting technology [2]. For Nb_3_Sn superconducting wires, the mainstream fabrication techniques include the bronze process [3], the internal tin process [4] and the powder-in-tube (PIT) method [5]. At present, both the bronze process and internal tin process have achieved industrial mass production. In contrast, the PIT method has several limitations. The preparation of the precursor NbSn_2_ powder involves complicated procedures and high technical barriers. Moreover, the required ultrafine high-purity copper powder and niobium tubes are expensive raw materials, which makes the overall manufacturing cost of the PIT method substantially higher than that of the other two processes and has hindered its large-scale industrial application.

For REBCO superconductors, early researchers attempted to fabricate YBa_2_Cu_3_O_7-δ_ (YBCO) wires and tapes using the PIT method. However, YBCO has a relatively short coherence length and suffers from severe weak-link behavior at grain boundaries, resulting in poor superconducting performance of the resulting conductors. Therefore, current REBCO superconductors are mainly processed into biaxially textured coated conductors by methods such as chemical solution deposition (CSD), metal–organic chemical vapor deposition (MOCVD), and pulsed laser deposition (PLD), and these processes increase production costs [6].

**Figure 1:**
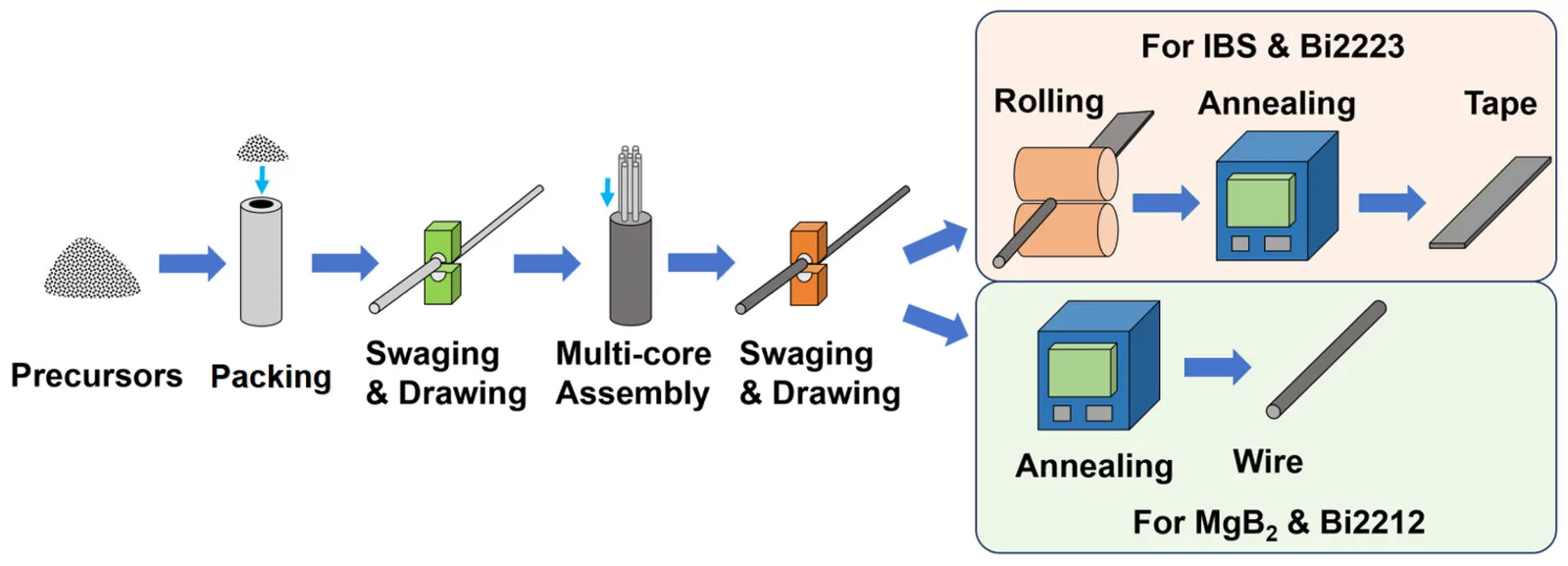
Schematic of the fabrication processes for superconducting wires and tapes by the PIT method.

BSCCO, MgB_2_, and IBS can all be fabricated into wires and tapes via the powder-in-tube method. Figure 1 illustrates the typical processing route for PIT-processed superconducting wires and tapes. Depending on the state of the precursor powder loaded into the metal tube, the PIT process can be divided into two categories: one is ex-situ PIT, in which the precursor powder is sintered to form the target superconducting phase before tube filling and deformation; the other is in-situ PIT, in which the elemental or alloyed starting materials are directly packed into the tube, and the superconducting phase is formed in-situ during the subsequent heat treatment of the wire.

Throughout the entire PIT fabrication process for superconducting wires and tapes, the quality of the precursor powder is the decisive factor governing the final performance of the product. Defects in the precursor powder, such as trace residual impurity phases, elemental segregation, abnormal particle size distribution, and elemental enrichment, can evolve continuously during subsequent mechanical processing and heat treatments, inducing increased porosity in the superconducting core and abnormal phase transformations, ultimately resulting in a degradation of the critical current density (*J*_c_). Particularly for the BSCCO system, the phase purity of the precursor powder is closely linked to liquid-phase reactions; for MgB_2_ and IBS, on the other hand, powder reactivity is intimately associated with sheath reactions, densification behavior, and grain connectivity. Most existing reviews have focused predominantly on the properties of superconducting wires and tapes, heat-treatment protocols, or application advances, while comparatively few have conducted cross-system comparisons using the chain of “precursor powder-core microstructure-wire performance” as the central theme. The present paper aims to compare the differences in precursor powder preparation routes across different superconductor families and their respective effects on wire and tape performance. We first propose a set of generic evaluation criteria for PIT precursor powders, then discuss the key controlling parameters specific to the BSCCO, MgB_2_, and IBS, and finally compare the common bottlenecks and differentiated solution strategies for industrial production among these three material classes.

To place the cross-system comparison on a more quantitative footing, Table 1 summarizes the principal production-cost and scalability indicators of the three PIT-processable conductor families. The systems differ markedly in sheath cost: Bi-based conductors must be sheathed in silver, the only chemically compatible and oxygen-permeable sheath material, and the silver content represents a dominant share of the total conductor cost, whereas MgB_2_ and Ba-122 wires can be fabricated with inexpensive Fe, Cu, or Nb sheaths. The heat-treatment windows also differ widely, from the partial-melting processing of Bi-2212 (approximately 830–890 °C), through the 580–900 °C doping windows of MgB_2_, to the approximately 750 °C sintering of Ba-122. In terms of powder scalability, kilogram-scale co-precipitation has been demonstrated for pilot-scale tape manufacturing [7], and spray pyrolysis offers the best industrial scalability owing to its compositional uniformity and batch consistency [8]; MgB_2_ relies on mature solid-state mixing and ball milling, whereas Ba-122 adopts a two-step solid-state route under strict inert-atmosphere protection [9]. These quantitative differences directly determine the manufacturing cost and length-consistency prospects of each system and motivate the system-specific discussions in Section 3, Section 4 and Section 5.

## 2. Fundamental Characteristics of High-Quality Precursor Powders for Superconducting Wires and Tapes

Based on the processing characteristics of the PIT method for fabricating superconducting wires and tapes, precursor powders generally need to possess the following fundamental characteristics:

The first requirement is that the precursor powders must have precise chemical composition and uniform elemental distribution. Specifically, the proportions of the major elements should closely match the target stoichiometry, and the dopant elements should be homogeneously distributed both within individual grains and along grain boundaries. Such uniformity helps prevent excessive local liquid phase, local compositional deficiency, and abnormal grain growth during the subsequent heat treatment. Taking IBS Ba_1−x_K_x_Fe_2_As_2_ (Ba-122) precursor powders as an example, the raw materials K and As are chemically active and tend to volatilize during high-temperature sintering, leading to elemental loss and the formation of impurity phases such as FeAs and metallic Fe particles. However, excessive addition of K and As can introduce residual free K and As impurities. Therefore, precise control of the K and As stoichiometric compensation is essential for regulating the impurity phases in Ba-122 precursor powders.

The second requirement is that the phase composition should be controllable. For most superconductors, the precursor powders are not required to be fully converted into the target superconducting phase. Instead, the initial phase assembly should be compatible with the subsequent phase evolution pathway. In the case of Bi_2_Sr_2_Ca_2_Cu_3_O_10+X_ (Bi-2223), specific intermediate phases or the two-powder route are commonly employed to regulate the reaction kinetics. By contrast, for MgB_2_ and Ba-122, either pre-synthesized superconducting phase powders or unreacted raw materials can be used.

The third requirement is that the powder particle characteristics need to be well controlled, including particle size, particle size distribution, morphology, degree of agglomeration, specific surface area, and flowability. These parameters collectively affect the powder packing density, the uniformity of deformation, and the degree of sintering densification. This effect is particularly significant for MgB_2_ wires and tapes fabricated by the in-situ PIT method. The chemical reaction between Mg and B induces volume shrinkage and Mg volatilization, which creates pores within the MgB_2_ superconducting core and consequently degrades the final tape performance. The use of nano-sized Mg powder can effectively reduce porosity and improve the tape performance.

The fourth requirement is that the precursor powders should exhibit good chemical compatibility and mechanical matching with the sheath materials. This serves to suppress detrimental interfacial reactions with Ag, Cu, Fe, and other sheath constituents, and to maintain microstructural stability during rolling, drawing, and heat treatment. For bismuth-based superconductors, silver is the only sheath material that does not react chemically with the superconductor and, at the same time, permits oxygen permeation at high temperatures. Accordingly, high-performance Bi-based superconducting wires and tapes universally adopt silver sheaths. For MgB_2_ and Ba-122 wires and tapes, the raw material ratios and synthesis procedures of the precursor powders must be tailored according to the selected sheath material, and the subsequent cold working and heat treatment schedules should be adjusted correspondingly.

In actual fabrication processes, different superconductors also have distinct requirements for the aforementioned characteristics. Bi-based superconductors feature complex phase formation mechanisms, so priority is given to controllable phase structure. For MgB_2_, the optimization of particle size and morphology is emphasized to reduce porosity and ensure uniform chemical doping. Since component loss and impurity phase formation easily occur in Ba-122 during post heat treatment, high precision of raw material composition is strictly required.

The four criteria differ greatly in importance across various systems. The primary bottleneck of Bi-based superconductors lies in the control of competing phase formation and phase purity [10]. Particle size and morphology optimization is the top concern for MgB_2_. Compositional accuracy represents the key challenge for Ba-122. To clearly illustrate the system-dependent dominant control factors and their specific influences on conductor microstructure and superconducting performance, the key processing parameters and their corresponding effects are summarized in Table 2. The following sections will discuss the dominant criteria based on the characteristics of each system.

It is worth emphasizing that the correlations among precursor characteristics, microstructure and superconducting performance constitute the central logic of the comparisons made in this review. Chemically, deviations in stoichiometry and impurity content are directly inherited by the reacted superconducting phase and dictate the formation of secondary phases; physically, the particle size distribution, morphology and agglomeration state of the precursor powder determine the packing density of the green body, which in turn controls the porosity and connectivity of the final conductor core; thermodynamically, the reactivity and phase-evolution pathway of the precursor define the achievable heat-treatment window. These multiscale correlations are demonstrated repeatedly for the three systems discussed below, and they justify the comparison criterion adopted throughout this manuscript: *J*_c_ and *J*_e_ values are compared under identical temperature and magnetic field conditions, and whenever the conditions differ, the measurement temperature and field are explicitly stated.

## 3. Bismuth-Based Superconductors

### 3.1. Classification and Characteristics of Bismuth-Based Superconductors

**Figure 2:**
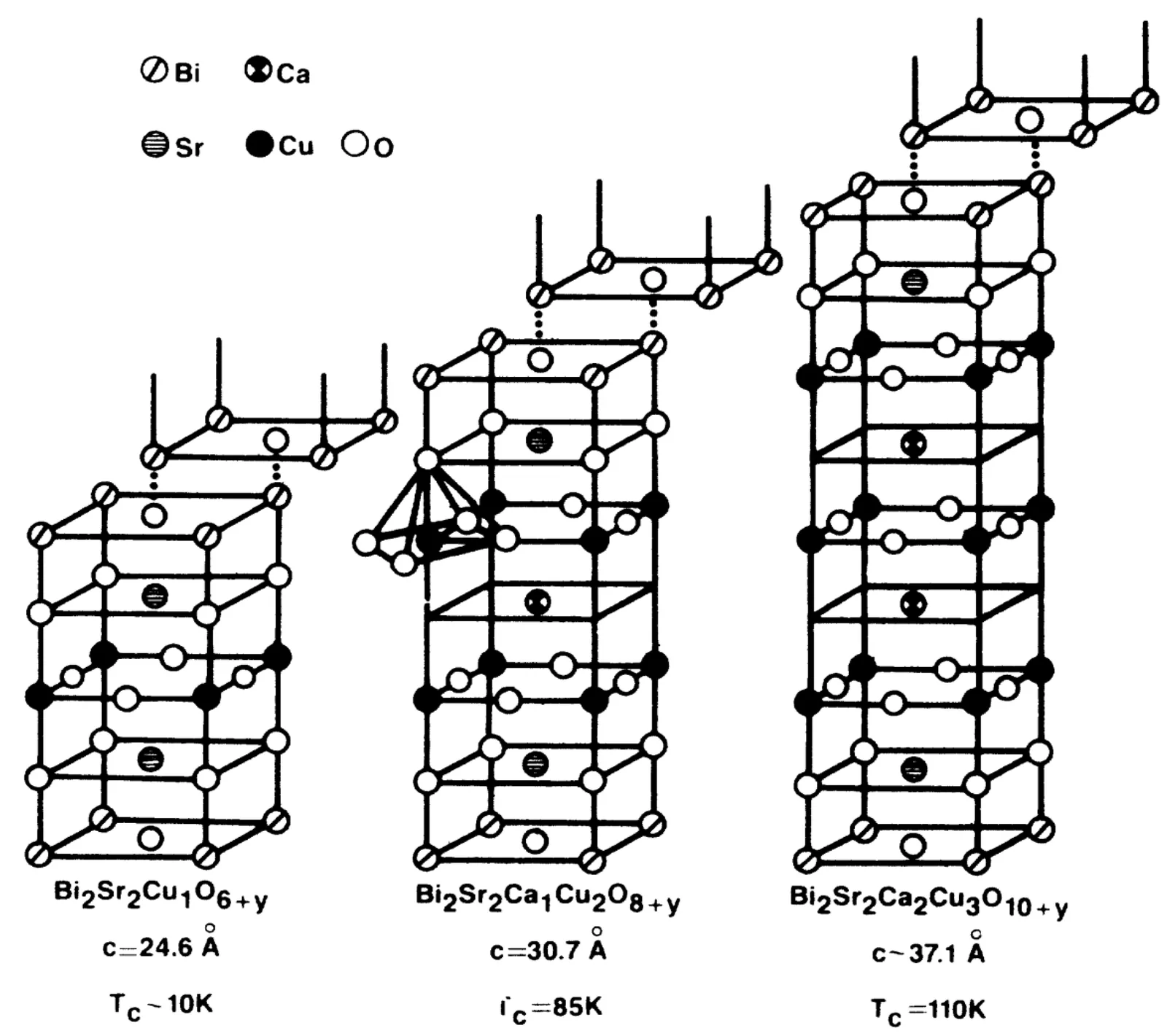
The crystal substructures of the Bi phases of general formula Bi_2_Sr_2_Ca*_n_*_-1_Cu*_n_*O_y_ with *n* = 1, 2, and 3 [14].

Bismuth-based superconductors generally fall into three prototypical phases: Bi_2_Sr_2_CuO_6+y_ (Bi-2201), Bi_2_Sr_2_CaCu_2_O_8+y_ (Bi-2212) and Bi_2_Sr_2_Ca_2_Cu_3_O_10+y_ (Bi-2223) [11,12,13], with their crystal structures as shown in Figure 2. Their critical temperatures (*T*_c_) are roughly 10, 85, and 110 K, respectively. Among these three phases, Bi-2212 and Bi-2223 have been the main focus for long-length wire fabrication, whereas the much lower *T*_c_ of Bi-2201 precludes the use of this material in practical power applications.

Bi-2212 sustains high current densities under large magnetic fields, despite its *T*_c_ of only 85 K; its appeal lies primarily in its high upper critical field [15]. At 4.2 K and 45 T, the engineering critical current density (*J*_e_) exceeds 200 A·mm^−2^. The processing route is also straightforward: well-connected grain networks can be achieved with a single heat-treatment cycle. Unlike other high-temperature superconductors, Bi-2212 can be extruded into uniform round wires without rolling deformation [16]. This round cross-section greatly simplifies the fabrication of insert coils and cable-in-conduit conductors (CICC), making Bi-2212 a leading candidate for fusion magnet systems currently under development.

Bi-2223 presents a different trade-off. It offers a higher *T*_c_ of 110 K and provides practical current densities at 77 K, with *J*_e_ typically around 10^4^ A·cm^−2^ in liquid nitrogen. Its plate-like grain morphology, however, imposes a processing constraint: mechanical rolling is required to align grains and reduce weak-link barriers for efficient current transport. Despite this added step, textured Bi-2223 tapes have found extensive use in power cables, fault-current limiters, motors, energy storage units, and high-field magnets [17,18,19,20,21,22].

### 3.2. Preparation and Phase Control of Bi-2212 Precursor Powders

**Figure 3:**
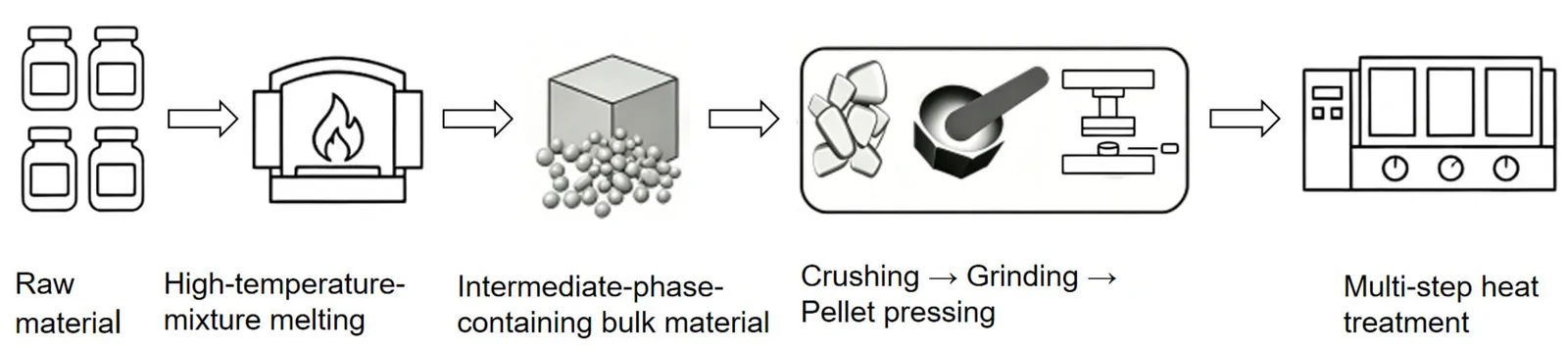
Flow chart of the solid-state reaction process.

There are various synthetic routes to prepare Bi-2212 precursor powders, among which solid-state reaction is the most conventional technique, alongside oxalate co-precipitation and spray pyrolysis. In the solid-state reaction method, raw materials are mixed and ground at stoichiometric ratios, followed by high-temperature calcination to form calcined agglomerates. After cooling, the agglomerates are crushed and reground; the obtained fine powders are pressed into pellets and subsequently treated via multi-stage heat treatment under flowing oxygen, as shown in the flow chart of the solid-state reaction process in Figure 3 [23]. Benefiting from simple operation and mature technical systems, this solid-state route was widely adopted in early syntheses of Bi-2212 and Bi-2223 superconductors. Nevertheless, oxide raw materials easily trigger elemental segregation, high impurity contents and uneven particle size distribution. In addition, repeated calcination cycles reduce production throughput. Accordingly, restricted by low productivity and poor batch-to-batch reproducibility, this route is unsuitable for large-scale commercial manufacturing.

**Figure 4:**
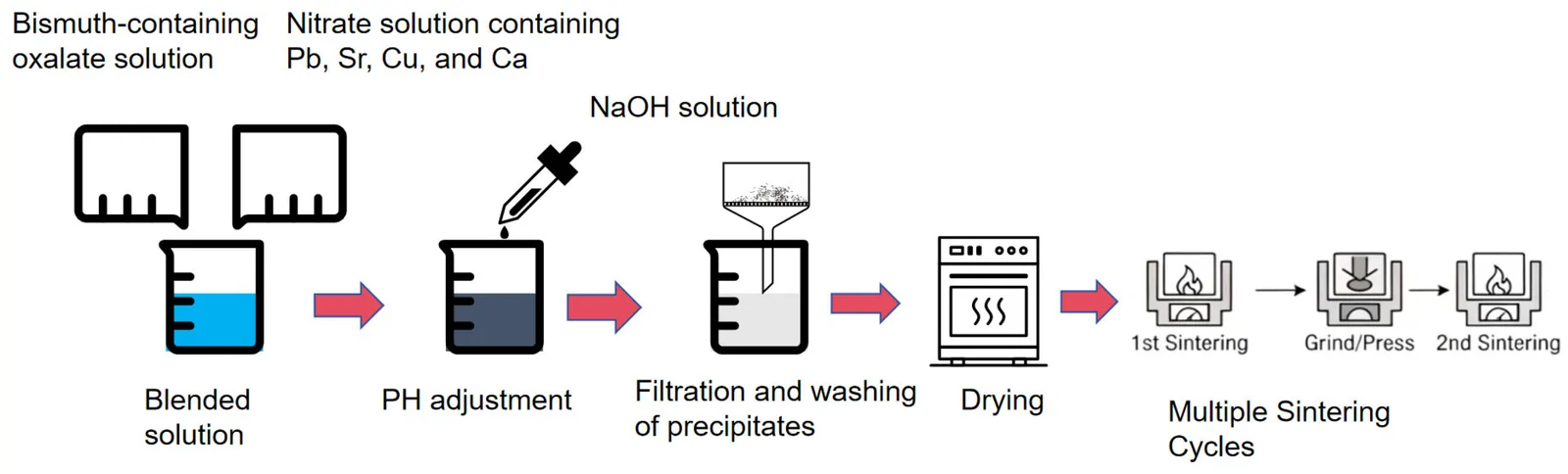
Process flowchart for the preparation of Bi-2212 precursor powders by co-precipitation method.

The oxalate co-precipitation method starts by dissolving raw metal salts in nitric acid, with oxalic acid added as the precipitant [24]. The pH value is tuned using alkaline solutions to induce coprecipitation, and the collected precipitates undergo multi-step calcination to obtain target precursor powders, as shown in the process flowchart for preparation of Bi-2212 precursor powders by co-precipitation method in Figure 4. This strategy produces particles with high reactivity and small sizes, while enabling precise regulation of carbon impurities. However, strict pH control and complicated operating procedures limit its scalability for industrial mass production.

**Figure 5:**
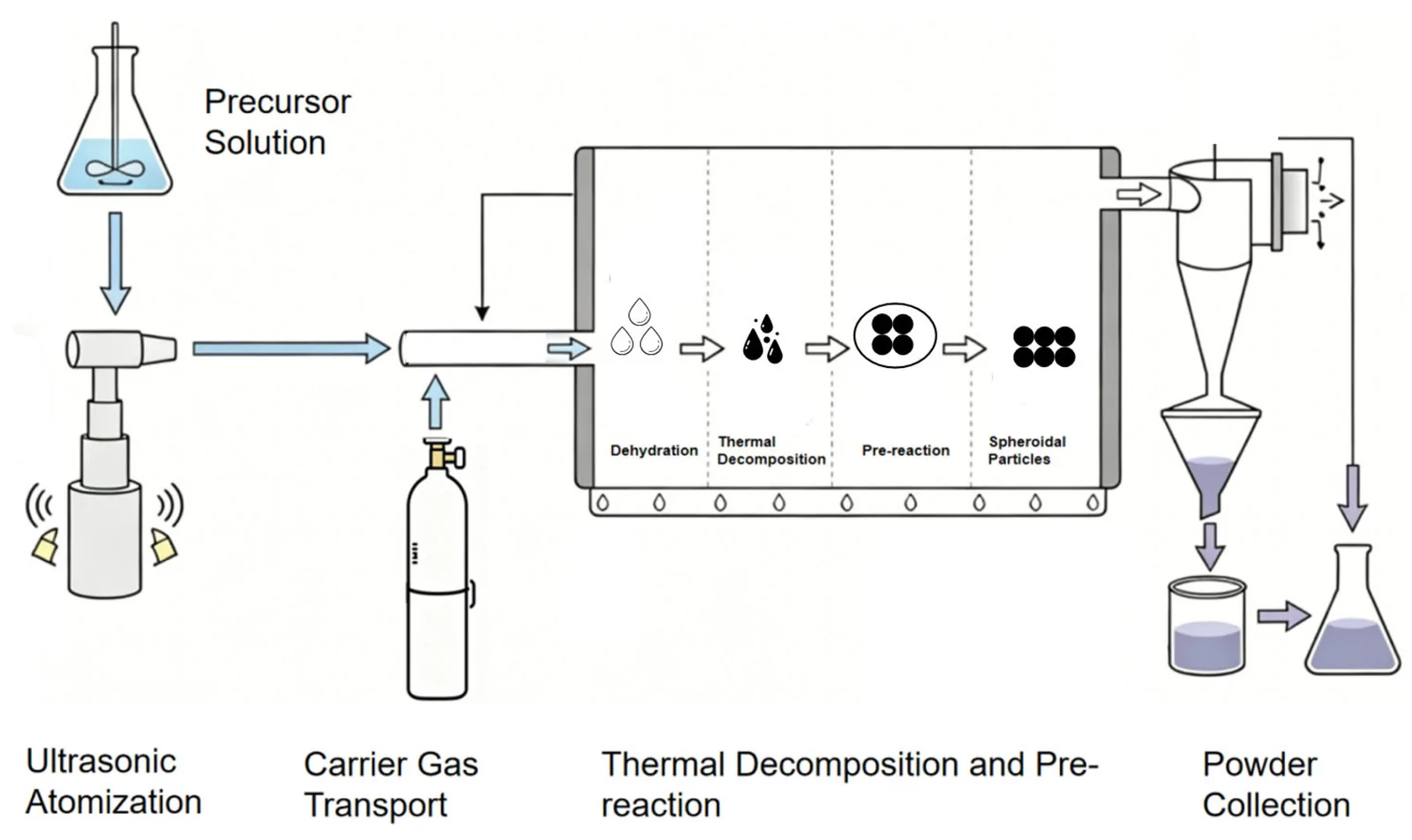
Schematic diagram of the stages of spray pyrolysis processes.

Spray pyrolysis has been adopted by enterprises including American Superconductor and Merck (Germany) for the industrial preparation of Bi-2212 and Bi-2223 precursor powders (Figure 5). This technology is highly suitable for mass production, as the as-synthesized powders exhibit uniform composition, fine particle size and excellent batch consistency. The fabrication procedure begins with ultrasonic atomization of mixed precursor solutions to form micro- and nano-sized droplets, which are transported into a high-temperature pyrolysis zone by carrier gas [24]. Inside the heating zone, droplets sequentially undergo dehydration, thermal decomposition and solid-state reactions to generate spherical particles for collection. The entire reaction completes within seconds to tens of seconds, realizing one-step conversion from solution to precursor powders and greatly improving production efficiency and powder quality. Even so, elemental volatilization loss occurs at high temperatures, which requires precise real-time temperature regulation.

Beyond classic fabrication routes such as solid-state reaction, oxalate co-precipitation and spray pyrolysis, Bi-2212 precursor powders can also be synthesized via spray drying [25], sol–gel method [26] and freeze-drying method [27]. Every processing route generates distinct secondary and impurity phases, so custom post-synthesis thermal treatments are mandatory to mitigate harmful byproducts. Even with optimized heat protocols, all these preparation methods encounter two core practical barriers: strong competitive phase formation and stringent requirements for impurity control.

A more direct approach has been to adjust the precursor stoichiometry. Nexans and Oxford Superconducting Technology carried out a series of large-scale industrial trials, adjusting the Sr/Ca atomic ratio from 1.34 to 2.25 [28,29]. Their testing identified Bi_2.17_Sr_1.94_Ca_0.89_Cu_2_O_x_ (named the “521” composition, Sr/Ca = 2.18) as the most promising precursor formulation. After overpressure heat treatment, wires made from this stoichiometry reached *J*_c_ of 7.6 × 10^5^ A·cm^−2^ measured at 4.2 K under self-field conditions.

This strong performance dependence on elemental ratios originates from complex phase equilibrium rules within the Bi-Sr-Ca-Cu-O multi-oxide system. Bi-2212 inherently contains multiple metallic cations, which means undesired secondary phases, such as alkaline-earth cuprate (AEC phase), CuO and Bi_x_(Sr,Ca)_y_O_z_, almost inevitably coexist alongside the target superconducting phase. This multiphase coexistence arises from two contributing factors. Bi-based superconducting phases share similar crystal frameworks and comparable formation activation energies, easily generating stacking faults and intergrowth defects during powder synthesis [30]. Meanwhile, non-superconducting AEC phases have reaction barriers equal to or lower than Bi-2212, and they exhibit significantly faster growth rates [31,32]. These thermodynamic and kinetic disparities create constant competition between the superconducting phase and parasitic impurities throughout the whole fabrication workflow, making single-phase Bi-2212 powder extremely hard to synthesize at scale [31].

Schulze et al. mapped phase stability boundaries for the Bi_x_(Sr_0.6_Ca_0.4_)_3-x_Cu_2_O_8_ system and confirmed that pure Bi-2212 only stabilizes within an extremely narrow Bi range of x = 2.03–2.07. Minor deviations from this window produce completely different phase assemblies. Insufficient bismuth drives precipitation of 2:1 AEC phase ((Sr,Ca)_2_CuO_3_) and free CaO, while excess Bi facilitates formation of the 11905 phase (Bi_2.2-x_Sr_1.8+x_Cu_5_O_z_) and CuO. Temperature also strongly influences the impurity-phase equilibria in liquid-solid coexistence regions. CuO dominates impurity assemblies near 850 °C, whereas the 14:24 AEC phase ((Sr,Ca)_14_Cu_24_O_y_) becomes dominant between 860 and 870 °C. At roughly 880 °C, both 2:1 AEC phase ((Sr,Ca)_2_CuO_3_) and 1:1 AEC phase ((Sr_1−x_Ca_x_)CuO_2_) emerge simultaneously; further temperature elevation fully decomposes the Bi-2212 superconducting matrix [33]. Such a narrow thermal processing window poses major challenges to consistent industrial production.

Given how difficult full impurity removal is to achieve, researchers have shifted their focus away from eliminating all secondary phases and toward controlled microstructural engineering to leverage beneficial minor phases. Naderi et al. uncovered a grain-size-dependent rule for Bi-2201 intergrowths via detailed microscopic characterization. Large Bi-2201 grains over 20 μm severely block charge transport and trigger weak-link behavior. However, nanoscale Bi-2201 intergrowths do not hinder current flow and instead serve as effective flux-pinning centers, raising *J*_c_ under high magnetic fields [34]. Therefore, ideal microstructures aim to eliminate coarse Bi-2201 domains while retaining dense nanoscale Bi-2201 intergrowths.

Multiple compositional tuning experiments have validated this microstructural design principle. Li et al. fabricated superconducting tapes using the standard 521 stoichiometry and a Ca-rich 524 alternative (Bi_2.14_Sr_1.66_Ca_1.24_Cu_1.96_O_x_) for direct comparison. Tapes derived from the 521 precursor delivered far higher *J*_c_ values. The improvement stems from two synergistic microstructural effects: the finely dispersed AEC particles and copper-free (CF) minor phases optimize the melt microstructure and reduce residual large current-limiting defects, thereby improving intergrain connectivity; meanwhile, the high-density nanoscale Bi-2201 intergrowths within the Bi-2212 grain colonies act as effective flux pinning centers. The combination of enhanced connectivity and stronger pinning collectively results in the improved performance [35]. In contrast, bulky Bi-2201 grains in the Ca-rich 524 sample impaired superconducting performance, matching the microscopic trends reported by Naderi et al. [34]. Li’s follow-up research isolated the individual impact of Sr/Ca stoichiometry. Wires prepared with Sr/Ca = 2.24 exhibited a *J*_c_ 1.5 times that of counterparts with Sr/Ca = 2.33. Mechanistic analysis reveals that Sr/Ca ratios directly modify the formation kinetics of intermediate 14:24 AEC. Since this intermediate phase dictates the overall solid-state reaction pathway for Bi-2212, regulating its generation improves final phase purity and charge transport performance [36]. Jin et al. took a direct phase-engineering approach by mixing 2 wt.% synthetic 14:24 AEC powder into precursor batches. The intentional addition suppressed large residual Bi-2201 grains and introduced abundant Bi-2201/Bi-2212 intergrowths and stacking faults inside the superconducting matrix. These synergistic microstructural features pushed the wire *J*_c_ up to 4.67 × 10^5^ A·cm^−2^ at 4.2 K under a 12 T applied field. The result solidifies the dual advantages of precise AEC phase regulation and nanoscale Bi-2201 flux pinning for high-field superconducting wires [37].

Apart from oxide secondary phases, trace non-metallic contaminants also degrade superconducting performance and cannot be overlooked. Shen’s group demonstrated that residual carbon and hydrogen tend to aggregate along grain boundaries, worsening weak-link effects and reducing the maximum current-carrying capacity of finished wires [38,39]. These residual impurities also induce void formation inside the wire matrix, offering an additional source of performance degradation beyond phase-related effects.

### 3.3. Phase Formation Mechanism and Composition Optimization of Bi-2223

Systematic phase-diagram studies of the Bi-2223 system reveal that without Pb addition, the superconducting phase exists only within a narrow temperature interval and remains in equilibrium with multiple secondary phases. The introduction of Pb results in aliovalent substitution at the Bi sites (Pb^2+^ replacing Bi^3+^), which necessitates charge compensation—for instance, through adjustments in oxygen stoichiometry or the Cu^2+^/Cu^3+^ mixed valence state—thereby modulating the carrier concentration in the CuO_2_ planes. This aliovalent substitution not only lowers the activation energy for Bi-2223 nucleation but also alters the reaction pathway; notably, Ca_2_PbO_4_ appears as a transient intermediate phase that participates in the formation of Bi-2223 rather than being merely suppressed. Consequently, the formation of the target phase is markedly accelerated, and its structural stability is enhanced. The resulting Pb-modified (Bi, Pb)_2_Sr_2_Ca_2_Cu_3_O_10+X_ (Bi-2223) exhibits improved superconducting performance, positioning it as a promising candidate for practical applications [40,41,42]. Early fabrication routes for Bi-Pb-2223 tapes were designed following the nominal cation ratio (Bi + Pb):Sr:Ca:Cu = 2:2:2:3. Nevertheless, a growing body of experimental evidence indicates that achieving high performance in high-*J*_c_ conductors requires intentional non-stoichiometric tuning. A typical strategy involves Sr deficiency coupled with moderate Ca and Cu excess; such chemical modification introduces abundant flux pinning defects and mitigates weak-link behavior between adjacent grains simultaneously.

Thermal stability constitutes another critical bottleneck during texture-forming sintering. As characterized in Ref. [43], the Bi-2223 phase is thermally fragile at temperatures near its peritectic melting threshold. Once the sintering temperature exceeds this threshold, thermal decomposition readily occurs, significantly reducing the fraction of superconducting Bi-2223 and deteriorating overall transport performance. Direct use of pure Bi-2223 powder as the starting precursor severely impairs the performance of PIT-processed tapes: intrinsically poor intergrain bonding drastically suppresses the achievable *J*_c_. A mature alternative precursor design relies on a Bi-2212 matrix blended with tailored Ca-Cu-O secondary powders. This chemical modulation adjusts the global cation stoichiometry to the target Bi-2223 ratio, driving in situ phase transformation via solid-state diffusion during sintering. Another critical design parameter is the controlled retention of trace Ca_2_PbO_4_ within the precursor. Thermal decomposition of Ca_2_PbO_4_ releases reactive Pb, which reacts with CuO to generate a transient liquid phase. This liquid medium serves two vital functions: it accelerates grain coarsening and alignment of Bi-2223, and it heals microstructural defects introduced by cold rolling [44]. Consistent with a body of prior work, Bi-2223 formation proceeds through a sequential two-step solid-state reaction path [45,46,47]:

Bi-2212 + Pb-containing phase → (Bi, Pb)-2212 + secondary phase

(Bi, Pb)-2212 + secondary phase → Bi-2223

Four mature synthetic techniques are widely employed for preparing Bi-2223 precursor powders: solid-state reaction, sol–gel synthesis, oxalate co-precipitation (CP), and aerosol spray pyrolysis (SP) [7,48,49,50]. For scalable industrial powder production, CP and SP have largely superseded conventional solid-state synthesis. Unlike solid-state mixing, their distinct particle-growth mechanisms enable precise regulation of phase formation during powder preparation. Cui’s team developed a kilogram-scale co-precipitation process capable of producing 3 kg of powder per batch for pilot-scale tape manufacturing [7]. Unfortunately, successive grinding operations inevitably trap carbon residues and disrupt cation stoichiometry. Carbon impurities form insulating carbonate compounds, inducing severe microstructural heterogeneity and poor *J*_c_ uniformity over long-length tapes. As an aerosol-based one-step synthesis, spray pyrolysis avoids mechanical grinding entirely, thus eliminating carbon pollution and stoichiometric drift to produce highly uniform precursor powders [8]. A side-by-side tape performance comparison conducted by Cui et al. shows that CP-derived tapes reach higher peak *J*_c_, whereas SP-derived precursors produce conductors with uniform transport properties along the entire length [7]. This advantage makes spray pyrolysis a more feasible choice for large-scale industrial manufacturing.

The dual-powder fabrication route was proposed to precisely control interphase solid-state reactions during Bi-2223 synthesis. In this approach, the synthesis of Bi-2212 and Ca-Cu-O (CCO, a mixture of Ca_2_CuO_3_ and CuO) is performed separately before mixing, so that the phase formation behavior of each component can be independently adjusted. Luo and coworkers systematically investigated the Pb partitioning factor z, which quantifies the fraction of Pb incorporated into Bi-2212. Their results demonstrate that Pb substitution into the Bi-2212 lattice yields orthorhombic grains and shortens the induction period for Bi-2223 nucleation. Nevertheless, full Pb doping within Bi-2212 (z = 0.4) fails to maximize the reaction rate at the early sintering stage. Free Ca_2_PbO_4_ containing residual Pb (z = 0.3) produces a liquid phase at low temperatures to accelerate cation diffusion, thereby delivering optimal initial reaction kinetics. Furthermore, samples with high z values exhibit elevated *J*_c_, attributed to the significant elimination of residual Bi-2212 impurities and intergrowth lattice defects [51].

Particle size has been established as a critical structural parameter for tuning the physicochemical properties of the CCO component in Bi-2223 precursor systems, as demonstrated by Zhang et al. By adjusting the pH during co-precipitation synthesis, CCO powders with different average diameters were successfully prepared. Excessively fine CCO particles were found to be detrimental to phase formation: when the mean particle size dropped below 0.75 μm, the interdiffusion reactions required for Bi-2223 grain growth were severely inhibited, leaving a substantial amount of unreacted secondary phases within the sintered bulk. In contrast, CCO powder with a moderate average diameter of 1.10 μm delivers optimized reaction kinetics. This particle size enables thorough and continuous conversion into pure Bi-2223 at a sintering temperature of 830 °C while effectively preventing thermal decomposition, yielding a high *J*_c_ exceeding 1.22 × 10^4^ A·cm^−2^ [52]. This size-dependent performance trend is further validated by investigations on the spray-pyrolysis-based dual-powder route. Relevant studies indicate that tapes fabricated with larger CCO particles exhibit a ~30% higher critical current (*I*_c_) than those employing finer CCO precursors. This enhancement originates from the suppression of late-stage phase decomposition, a common defect induced by overly rapid phase transformation during high-temperature sintering [53].

Beyond chemical composition tuning, thermal treatment and powder pretreatment parameters are equally critical for precursor quality. Zhang et al. revealed that premature Bi-2223 nucleation during precursor calcination leads to an ultrafine grain microstructure. Excessively refined grains aggravate intergranular weak links, significantly suppressing superconducting current transport. Accordingly, the calcination temperature must be kept below the threshold for spontaneous Bi-2223 formation to preserve an optimized precursor microstructure [50]. In addition to thermal conditions, powder grinding procedures also affect final tape performance. Liu et al. compared manual and mechanical grinding techniques and verified that mechanical milling effectively refines particle size, increases tap density, and improves batch uniformity. This pretreatment completely eliminates residual Bi-2201 impurities, thereby boosting the tape *I*_c_ from 86 A to 127 A [54].

The calcination atmosphere, dominated by oxygen partial pressure (p_O2_), governs phase evolution by modulating the valence state of Pb. Quantitative XPS characterization by Zhang et al. shows that increasing p_O2_ from 1.0% to 10.0% drastically lowers the Pb^2+^/Pb^4+^ ratio. Higher oxygen levels oxidize Pb^2+^ to Pb^4+^, which preferentially forms Ca_2_PbO_4_. Consequently, less Pb^2+^ remains available for substitution into Bi-2212, and secondary phases such as AEC and CuO grow into larger grains. Sintering at 790 °C in a 7.5% O_2_-Ar mixed gas provides the optimal precursor structure, achieving a tape *J*_c_ of 3.3 × 10^3^ A·cm^−2^. This performance surpasses that of samples treated under either 1.0% or 10.0% p_O2_ [55].

Overall, Bi-based precursor development has progressed from simple grain-size reduction and carbon-removal goals to full-process optimization, including synthesis route, dual-powder matching, grinding homogenization, and calcination conditions. Spray pyrolysis offers the best industrial scalability due to its excellent compositional uniformity and batch consistency, gradually replacing coprecipitation for mass production. The dual-powder strategy, which enables independent control of Bi-2212 and Ca-Cu-O phases, has become mainstream for Bi-2223. Key processing parameters include pH-adjusted CCO particle size, narrow temperature and oxygen-partial-pressure windows to prevent Bi-2223 decomposition, and mechanical milling for powder uniformity. For both Bi-2212 and Bi-2223, the overarching principle is that precise regulation of intermediate phase kinetics, rather than pursuit of absolute single-phase purity, dictates final wire performance. Compared with solid-state reaction (poor reproducibility) and single-powder routes (insufficient grain coupling), spray pyrolysis combined with the dual-powder approach provides the best balance for industrial production.

## 4. MgB_2_ Precursor Powders

### 4.1. Properties and Application Fields of MgB_2_

**Figure 6:**
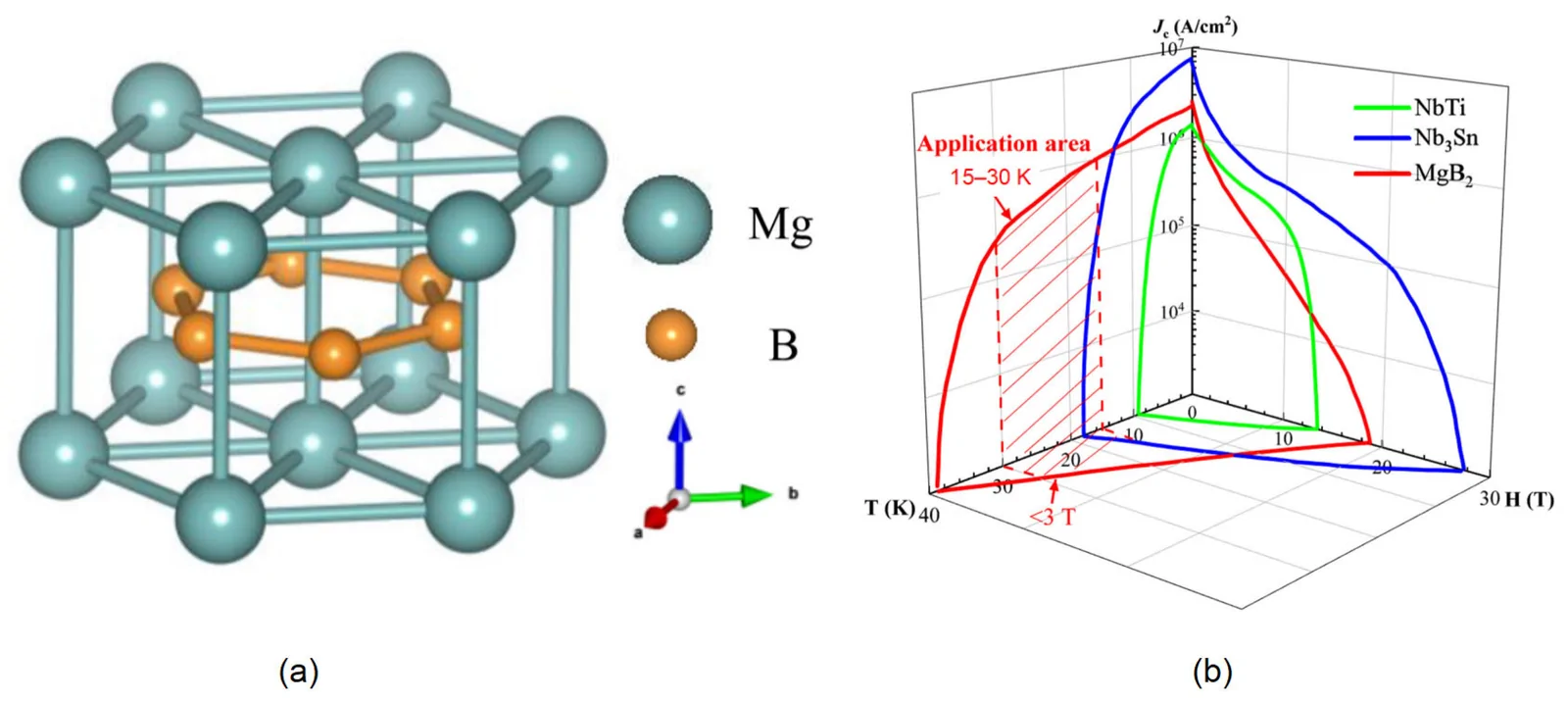
(**a**) Hexagonal crystal structure of MgB_2_. (**b**) Performance comparison between MgB_2_ and conventional superconductors NbTi and Nb_3_Sn. MgB_2_ shows optimal performance in low-fields at 20 K [59].

Since its discovery in 2001, MgB_2_ has attracted lasting interest as a practical intermetallic superconductor [56]. With a *T*_c_ of 39 K, approaching the McMillan limit, MgB_2_ exhibits the highest critical temperature among currently viable intermetallic compounds. Compared with conventional alloys such as NbTi and Nb_3_Sn, this higher *T*_c_ offers a significant engineering advantage: it permits operation with cryocoolers or liquid hydrogen, dramatically reducing overall system cooling costs as shown in Figure 6, which displays the hexagonal crystal structure of MgB_2_ and a performance comparison between MgB_2_ and conventional superconductors NbTi and Nb_3_Sn, where MgB_2_ shows optimal performance at 20 K in low magnetic fields.

In contrast to high-temperature oxide superconductors including REBCO and Bi-based systems, MgB_2_ is free from grain boundary weak-link issues. Its coherence length reaches 6–7 nm, and effective flux pinning centers can be introduced into the lattice through elemental doping to improve the current-carrying performance under high magnetic fields. Meanwhile, MgB_2_ exhibits low anisotropy, allowing efficient current transmission without elaborate texture control. In addition, its simple fabrication process makes it suitable for large-scale production of long wires. Owing to these advantages, MgB_2_ holds broad potential for practical applications in magnetic resonance imaging, superconducting power transmission cables, superconducting magnetic energy storage, magnets for large scientific facilities, and aerospace applications [57,58,59].

MgB_2_ exhibits excellent superconducting performance in low magnetic fields, where *J*_c_ values in the temperature range of 20–30 K meet practical application requirements.

However, its *J*_c_ degrades drastically at high fields, which restricts its application in high-field environments. To address this issue, numerous modification studies have been conducted to enhance the high-field superconducting performance of MgB_2_ [60]. Current research directions mainly focus on optimizing the microstructure, improving intergranular connectivity, and strengthening flux pinning at grain boundaries.

The fabrication of high-quality MgB_2_ precursor powders is a prerequisite for constructing high-performance MgB_2_ superconducting wires. During the in-situ synthesis of MgB_2_, the reaction between magnesium and boron is accompanied by a theoretical volume shrinkage of ~23.3% along with severe Mg volatilization, which inevitably generates interior pore defects within the superconducting core. These pores not only reduce the effective current-carrying cross-section but also break the continuity of the MgB_2_ matrix, thereby significantly suppressing *J*_c_. Unlike cuprate high-temperature superconductors that suffer from pronounced grain boundary weak-link effects, MgB_2_ exhibits negligible weak-link behavior at low temperatures; instead, its grain boundaries can serve as effective flux pinning centers [61]. Benefiting from the relatively large coherence length of MgB_2_, extrinsic lattice defects and secondary phases introduced by elemental doping can further act as additional pinning sites, leading to prominent *J*_c_ enhancement. Consequently, current performance optimization strategies for MgB_2_ wires converge on two core objectives: eliminating interior porosity to improve matrix densification and constructing high-density flux pinning centers. To circumvent the density limitations of the conventional PIT technique, the Internal Magnesium Diffusion (IMD) method has been developed as an innovative fabrication route, which significantly improves core densification and elevates transport properties. Nevertheless, the adoption of solid magnesium rods as the Mg source in IMD poses inherent challenges in mechanical forming and industrial processing; unoptimized IMD processes often result in poor axial structural uniformity and low fabrication repeatability for long-length wires, which restricts large-scale industrial production [59]. Given these practical constraints, PIT remains the dominant technology for mass fabrication. Although the IMD route avoids the direct use of precursor powders, it shares an equally stringent requirement for magnesium source purity with the PIT method. This intrinsic correlation between Mg purity and microstructure evolution provides valuable guidance for optimizing precursor compositions and regulating process parameters in industrial PIT fabrication.

### 4.2. Raw Materials for MgB_2_ Precursor Powders

Systematic investigations have established that the microstructure and quality of precursor powders critically influence both the porosity evolution and the final superconducting performance of MgB_2_ wires. Key powder parameters-specifically, powder type, chemical purity, particle size distribution, morphological uniformity, and residual oxygen content-are closely intertwined with the current-carrying capacity. Among these, residual oxygen and particle size have been identified as the most consequential factors, as they directly affect the reaction kinetics and impurity phase formation. To date, substantial research efforts have therefore been devoted to the optimization of the two fundamental raw materials: magnesium and boron powders.

Magnesium is highly reactive and readily oxidizes to MgO during storage and raw material preparation. The MgO impurities segregated at MgB_2_ grain boundaries act as resistive barriers that impede superconducting current transport, leading to deteriorated current-carrying performance. To mitigate oxide contamination, inert-atmosphere pretreatment is commonly implemented, while controlled ball milling is adopted to refine particle size and mechanically activate precursor powders. Importantly, ball milling cannot eliminate native oxide layers, which are generally removed via high-temperature reduction or chemical etching. Although magnesium hydride (MgH_2_) has been explored as a direct replacement for conventional Mg powder, this strategy fails to deliver satisfactory superconducting performance in fabricated tapes. Instead, existing studies confirm that MgH_2_ acts as a high-efficiency functional dopant for MgB_2_ microstructure modulation and superconducting property enhancement. The enhanced performance originates from the thermal decomposition of MgH_2_ during heat treatment: the released hydrogen suppresses Mg volatilization and reduces residual B_2_O_3_ impurities, while in-situ generated fine MgO particles serve as effective flux pinning centers to further enhance current-carrying capacity [62,63].

**Figure 7:**
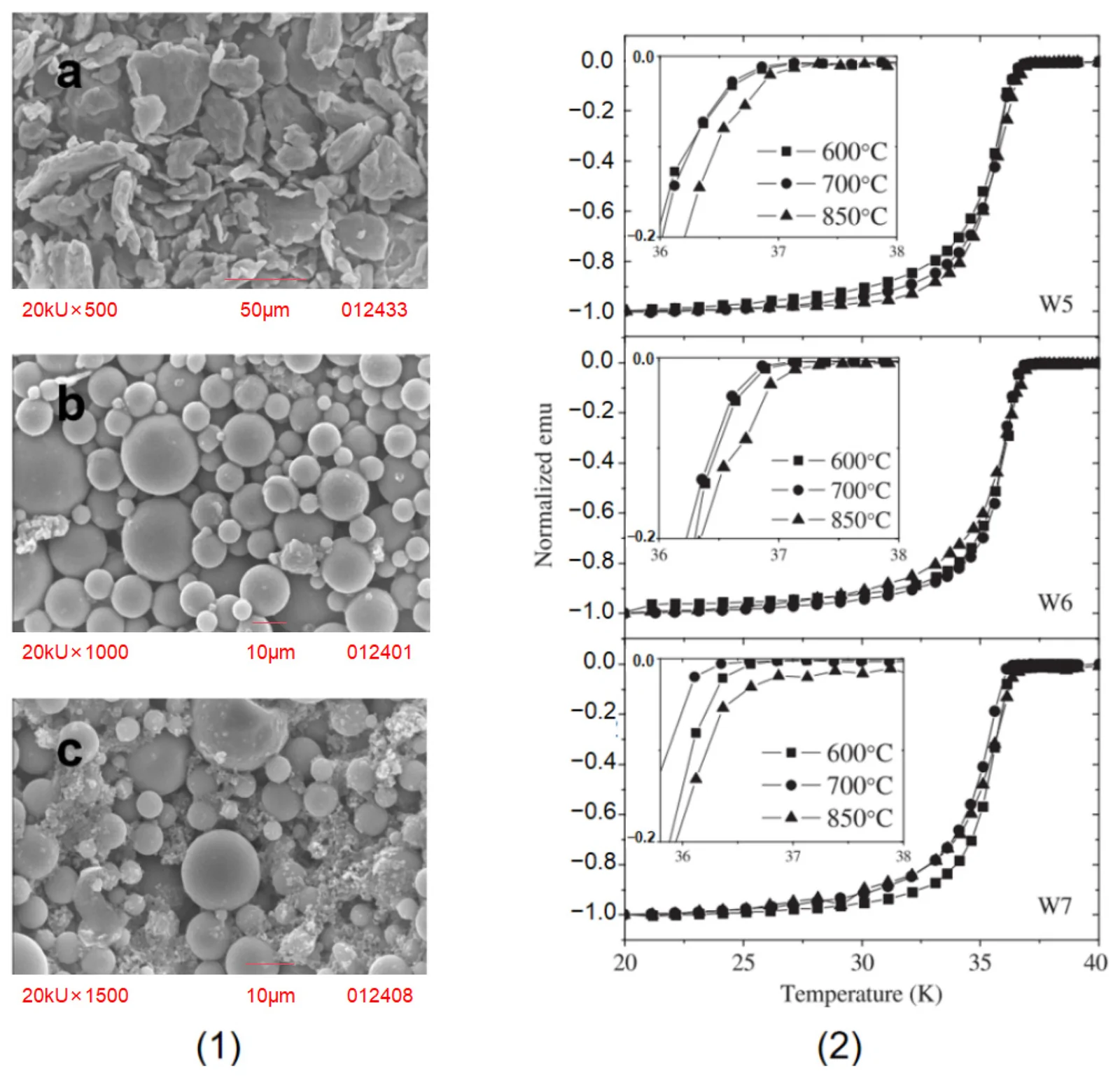
(**1**) SEM images of the magnesium powders with a particle size of (**a**) 44 μm (Alfa Aesar), (**b**) 12–17 μm (Tangshan Weihao), (**c**) 4–6 μm (Tangshan Weihao); (**2**) Magnetic moment measurements as a function of temperature for samples W5 (Magnesium powder size 44 μm), W6 (Magnesium powder (size 12–17 μm)) and W7 (Magnesium powder (size 4–6 μm)) annealed at 600 °C for 30 h, 700 °C for 30 min and 850 °C for 30 min [64].

In addition to chemical purity, the particle size of magnesium powder critically dominates the porosity and grain connectivity of final MgB_2_ superconducting wires. The pore evolution mechanism in MgB_2_ systems originates from the coupled effects of Mg-B in-situ reaction, precursor apparent density, and plastic deformation homogeneity. Specifically, the coalescence of lattice vacancies-arising from unbalanced Mg and B diffusion rates during sintering (Kirkendall effect)-combined with the intrinsic volume shrinkage (~23.3%), constitutes the primary sources of interior pore defects. Accordingly, grain refinement of Mg powder serves as an effective strategy to suppress pore generation and optimize inter-grain connectivity. Tan et al. systematically investigated the influence of Mg particle size on wire performance using Mg powders with different particle sizes. The morphological features of these graded Mg powders and the corresponding superconducting transport properties of fabricated tapes/wires are illustrated in Figure 7. Under identical heat treatment protocols, refined Mg powder contributes to reduced porosity, improved microstructural homogeneity, and enhanced superconducting performance [64]. Further studies by Maeda et al. verified that the graded mixing of coarse and fine Mg powders enables precise regulation of phase composition and pore structure [65]. Additionally, the introduction of excess Mg has been proven to optimize grain connectivity and elevate the critical current density of MgB_2_ wires [66]. Apart from Mg raw materials, the morphological characteristics and particle size distribution of boron powder also profoundly determine the microstructure and superconducting performance of MgB_2_ superconductors. Maeda et al. fabricated MgB_2_ wires using variable-sized B powders coupled with fixed coarse Mg powder, confirming that fine B powders facilitate microstructure optimization and improve current-carrying capability [67]. In contrast, coarse crystalline B powders are prone to severe agglomeration, leading to localized elemental segregation, reduced sintering density, and suppressed critical current performance [68]. Compared with crystalline B powder, amorphous B powder possesses an isotropic lattice structure and high specific surface area, delivering significantly higher reaction activity and superior superconducting performance in final tapes [69]. An alternative approach to mitigate reaction-induced porosity involves the use of MgB_4_ as a precursor. Theoretical evaluations reveal that the Mg-MgB_4_ reaction exhibits a low volume shrinkage of only ~15%, which substantially reduces pore formation. Benefiting from the optimized pore structure, the modified MgB_2_ samples exhibit a critical current density of 7.8 × 10^4^ A·cm^−2^ at 20 K and 2 T [70].

From a conductor perspective, the most useful way to connect these powder parameters to performance is through the active cross-sectional fraction, that is, the fraction of the core area that actually carries current. In in-situ PIT wires, this fraction is reduced both by porosity and by the insulating phases, mainly MgO and B_2_O_3_, that decorate the grain boundaries, and both are inherited largely from the raw materials rather than created during deformation [62,63,71]. This is why the purity of the boron source, and in particular its oxygen content, has a disproportionate influence on *J*_c_: it acts simultaneously on the pore fraction, on the grain-boundary transparency and on the effective incorporation of carbon.

Recent work on low-purity B in L-IMD MgB_2_/Fe wires prepared with a coarse 98% Mg precursor found that the 86.7 wt% B sample showed partially improved *T*_c_, lower MgO/Fe_2_B fractions and higher magnetic *J*_c_ than the 95.2 wt% B sample, while its transport *J*_c_ remained comparable to literature values; however, because the B powders also differed in Mg impurity content, the effect of B purity alone was not isolated [72]. In SiC-doped PIT MgB_2_ wires measured between 15 and 30 K, transport *J*_c_ was influenced by SiC content, annealing conditions, grain shape and the two-region core morphology: 2 at.% SiC wires with point-dominant pinning showed higher transport *J*_c_ than 6 at.% SiC wires with surface-dominant pinning, and the effect of the two-region morphology was temperature- and SiC-content-dependent rather than uniformly controlling transport *J*_c_ over the whole 15–30 K range [73].

### 4.3. Chemical Doping of MgB_2_

The relatively large coherence length of MgB_2_ provides ample spatial tolerance for incorporating nanoscale secondary phases, making chemical doping a particularly effective strategy for enhancing flux pinning behavior and improving the critical current density, especially under high magnetic fields. In a seminal systematic study, Collings et al. classified the performance enhancement mechanisms of dopants in MgB_2_ systems into four primary categories [74].

First, lattice substitution effects: partial atomic substitution can occur at either Mg or B sites. Substitution on Mg sites generally deteriorates superconducting properties, whereas carbon substitution on B sites effectively enhances the high-field critical current density and elevates the upper critical field.

Second, construction of effective flux pinning centers: dopant-induced secondary phases with characteristic dimensions smaller than the coherence length of MgB_2_ can serve as powerful flux pinning sites, restraining magnetic flux creep and further improving the current-carrying capacity.

Third, microstructural grain refinement: introduced dopants inhibit excessive grain growth during sintering, reducing the average grain size. The resultant abundant grain boundaries provide denser pinning sites and significantly strengthen grain-boundary flux pinning.

Finally, optimization of intergranular connectivity: rational doping reduces residual porosity, promotes uniform phase distribution, and effectively improves grain-to-grain connectivity within the MgB_2_ matrix, ultimately contributing to a comprehensive enhancement in critical current density.

**Figure 8:**
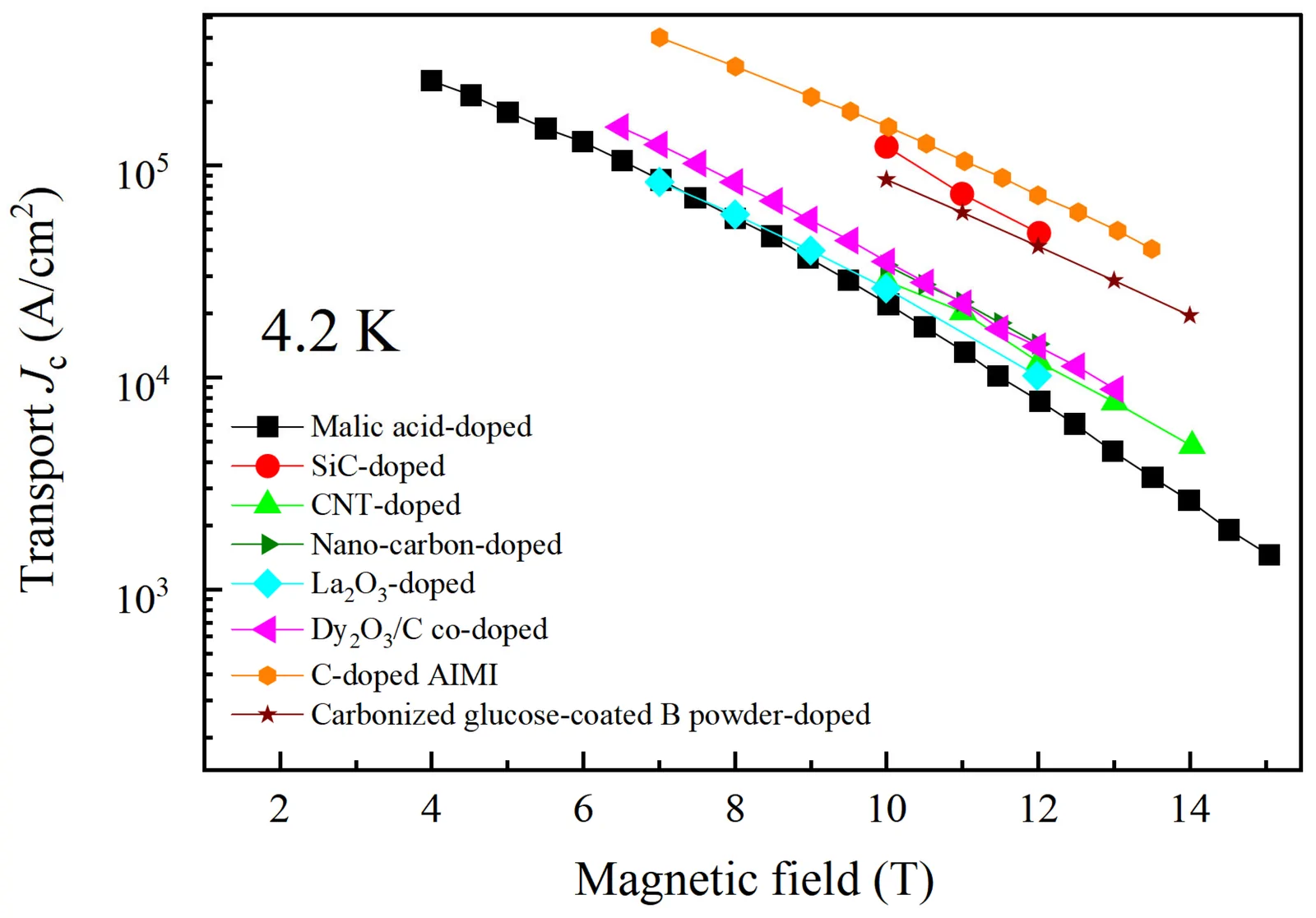
Critical current density (*J*_c_) versus magnetic field (H) curves at 4.2 K for MgB_2_ wires/tapes with different dopants. The data refer to Malic acid-doped [75], CNT-doped [76], Nano-carbon-doped [82], C-doped AIMI (advanced internal magnesium infiltration) [83], La_2_O_3_-doped [78], Dy_2_O_3_/C co-doped [79] and Nd_2_O_3_-doped [80]. Data source for SiC-doped and Carbonized glucose-coated B powder-doped samples: Institute of IEE CAS.

In practice, most dopants enhance superconducting performance via the synergistic coupling of multiple mechanisms outlined above. Nevertheless, chemical doping still presents inherent limitations, including inhomogeneous dopant distribution, dopant aggregation at grain boundaries that degrades intergranular connectivity, and increased impurity levels within the MgB_2_ matrix. To systematically evaluate these trade-offs, current doping strategies are broadly categorized according to dopant chemistry into three groups: carbon-based, metallic, and oxide doping. The effectiveness of these strategies is illustrated in Figure 8, which compares the critical current density (*J*_c_) versus magnetic field (H) curves at 4.2 K for MgB_2_ wires/tapes doped with various representative dopants, including malic acid, SiC, CNT, Carbonized glucose-coated B powder, La_2_O_3_, Dy_2_O_3_/C, and Nd_2_O_3_ [75,76,77,78,79,80]. These data reveal marked differences in the field-dependent *J*_c_ behavior among the different doping categories, providing a direct experimental basis for evaluating the trade-offs mentioned above.

In particular, an inhomogeneous dopant distribution gives rise to a spatially non-uniform *J*_c_ along the conductor: underdoped regions remain weakly pinned and can become weak links limiting high-field transport, whereas locally overdoped regions may be accompanied by excess secondary phases that degrade intergranular connectivity. Since the engineering current density of a long wire is limited by its weakest cross-section, such dopant non-uniformity can constitute an important source of batch-to-batch variability, closely analogous to the agglomeration problem reported for coarse crystalline boron powders [68] and consistent with the microstructural observations on IMD MgB_2_ wires by Rosová et al. As noted by Gao et al. in their review of practical doped MgB_2_ conductors, dopant homogeneity remains a key issue; nano-sized dopants offer a larger specific surface area and, in principle, better dispersibility, but they cannot by themselves completely avoid agglomeration. The high surface energy of nanoparticles favors re-agglomeration during mixing, milling, and storage, and agglomerates far exceeding the MgB_2_ coherence length (6–7 nm) lose their function as effective point pinning centers. Effective nano-doping strategies therefore need to couple dopant selection with dispersion-promoting process steps, such as liquid-phase mixing of hydrocarbon dopants [81], carbonized glucose-coated boron powders, and optimized ball-milling parameters, to break up agglomerates and stabilize a homogeneous dispersion. Even with these measures, complete suppression of local aggregation remains difficult at the industrial scale and is one of the important origins of batch-to-batch performance fluctuation.

Among these, carbon doping has been the most extensively investigated since the initial discovery of MgB_2_ superconductivity [71,84,85,86]. It is well established that substitution of B sites by C atoms effectively improves the upper critical field and high-field critical current density of MgB_2_ superconductors. Common carbon sources for MgB_2_ doping mainly include nanocarbon, nano-SiC, and hydrocarbon-based precursors. Effective incorporation of nanocarbon and nano-SiC generally requires sintering temperatures ranging from 800 to 900 °C. Such high-temperature sintering inevitably induces excessive grain coarsening, promotes the formation of additional MgO impurities, and leaves residual carbon. The residual carbon tends to segregate at grain boundaries, severely degrading intergranular connectivity and impairing transport properties. Despite these drawbacks, nanocarbon doping can still significantly enhance high-field performance, enabling nanocarbon-doped MgB_2_ tapes to achieve a critical current density of 1.85 × 10^4^ A·cm^−2^ at 4.2 K and 10 T [87]. In addition, the MgB_2_ wire fabricated by the Institute of Electrical Engineering, Chinese Academy of Sciences (IEE CAS) using magnesium rods (φ3.0 mm, 99.95%), high-purity crystalline boron powder (4 μm, 99.999%), and 7 at% nano-SiC powder (20–30 nm, 99%) as raw materials exhibits a *J*_c_ of 1.23 × 10^5^ A·cm^−2^ at 4.2 K and 10 T, representing the state-of-the-art performance among SiC-doped samples reported to date. In contrast, hydrocarbon dopants can be uniformly dispersed in precursor powders via liquid-phase mixing and sintered at a relatively low-temperature window of 580–660 °C, effectively suppressing grain coarsening. However, partial thermal decomposition of hydrocarbons during heat treatment releases gaseous by-products, which introduce microdefects and weaken intergranular bonding. For malic acid (C_4_H_6_O_5_)-doped MgB_2_ tapes, a peak critical current density of 3.0 × 10^4^ A·cm^−2^ is achieved at 5 K and 8 T [81]. Significantly, MgB_2_ tapes doped with 4-dimethylaminobenzaldehyde (C_9_H_11_NO) exhibit an improved critical current density of 3.8 × 10^4^ A·cm^−2^ at 4.2 K and 10 T [88]. On the other hand, the IEE CAS further enhanced the performance of MgB_2_ wires by using carbonized glucose-coated boron powder as a carbon source. The maximum *J*_c_ reached 0.9 × 10^5^ A·cm^−2^ (4.2 K and 10 T), and *J*_e_ reached as high as 1.3 × 10^4^ A·cm^−2^ (4.2 K and 10 T). In terms of *J*_c_, a gap remains compared with the highest *J*_c_ reported to date [83]; however, in terms of *J*_e_, it has reached the internationally leading level.

Unlike carbon doping, which predominantly occurs at B sites, metallic doping enables performance modulation through Mg-site lattice substitution, intergranular connectivity optimization, and the construction of novel flux pinning centers. Experimental studies have shown that only a limited number of metallic elements-including aluminum, chromium, and lithium-can achieve partial Mg-site substitution within the MgB_2_ lattice. Among these substitutable dopants, moderate Al doping contributes to improved critical current density, whereas both Cr and Li doping monotonically degrade superconducting performance over conventional doping ranges. Specifically, Al doping below 2.5 at.% effectively enhances the upper critical field; however, excessive Al incorporation beyond 5 at.% induces lattice distortion and structural deterioration, leading to a general degradation of superconducting properties. Although Cr and Li are thermodynamically capable of Mg-site substitution, their doping introduces detrimental lattice defects and suppresses superconductivity at all tested concentrations.

In contrast to substitution-type metallic doping, titanium doping does not trigger lattice substitution. Instead, in-situ-generated TiB_2_ secondary phases serve as grain refiners to refine the microstructure and improve intergranular bonding, thereby optimizing current transport behavior [89]. Similarly, ZrB_2_ precipitation induced by Zr doping effectively tailors flux pinning efficiency and elevates the superconducting performance of MgB_2_ wires [90]. Among these metallic dopants, Ti and Zr have been identified as particularly efficient in simultaneously optimizing microstructural homogeneity, grain connectivity, and low-field critical current density. Notably, although Al doping offers a narrow optimal window (≤2.5 at.%) for enhancing the upper critical field, its narrow processing margin and structural deterioration beyond 5 at.% severely limit practical applicability.

In oxide doping systems, endogenous MgO impurities in raw precursor powders are generally regarded as detrimental to the superconducting performance of MgB_2_ wires. Such performance degradation is primarily attributed to the presence of large-scale, severely agglomerated MgO particles, which disrupt matrix continuity and block superconducting current transport. In sharp contrast, uniformly dispersed trace nano-MgO particles can effectively optimize the microstructure and superconducting properties. Jiang et al. verified that low-dose MgO addition (below 2.5 wt.%) significantly improves intergranular connectivity; simultaneously, the uniformly distributed nano-MgO particles function as extrinsic flux pinning centers, thereby enhancing the critical current density of MgB_2_ composites [91]. Beyond MgO doping, rare earth oxide doping has also been proven to introduce high-efficiency pinning sites and optimize flux pinning behavior [92]. Among various rare earth oxides, Y_2_O_3_ and CeO_2_ are the most widely investigated dopants for MgB_2_ modification. Their incorporation effectively restrains excessive grain growth during sintering and achieves refined microstructures, which synergistically improve intergranular connectivity and critical current density. Such performance enhancement originates from the in-situ formation of nanoscale rare-earth-rich secondary phases, which serve as robust flux pinning centers to suppress flux creep and optimize superconducting transport properties.

Further experimental investigations on rare-earth oxide doping have confirmed their significant beneficial effects on high-field superconducting performance. Yao et al. demonstrated that MgB_2_ tapes doped with 5 at.% Nd_2_O_3_ achieved optimized high-field critical current density, attributed to the in-situ formation of nano-sized NdB_6_ particles that serve as effective flux pinning centers [93]. Similarly, Zhang et al. incorporated nano-La_2_O_3_ into MgB_2_ precursors and confirmed that MgB_2_ wires doped with 5 wt.% La_2_O_3_ exhibited a nearly twofold enhancement in *J*_c_ at 4.2 K and 8 T relative to undoped counterparts [78]. This mechanism parallels that of Nd_2_O_3_ doping: the dispersed nano-La_2_O_3_-derived secondary phases introduce abundant volumetric flux pinning sites to suppress high-field flux creep. To further combine multiple modification effects, Wan et al. explored the combined effects of C/Dy_2_O_3_ co-doping on the microstructural and superconducting properties of MgB_2_ wires. Their results revealed that C/Dy_2_O_3_ co-doping simultaneously promotes carbon substitution at B sites and introduces Dy-containing pinning centers, yielding a synergistic enhancement in critical current density that exceeds either single-dopant system alone [94].

In summary, chemical doping effectively enhances MgB_2_ wire performance through lattice substitution, flux pinning, grain refinement, and connectivity improvement. Carbon doping boosts high-field *J*_c_ via B-site substitution; metallic doping strengthens grain boundaries and low-field performance; oxide doping introduces nanoscale pinning sites. Proper dopant selection and dosage are crucial to suppress impurities and segregation. Beyond doping, precursor morphology (including Mg particle size, B amorphicity, and dopant dispersion) governs porosity and connectivity. Unlike Bi-based systems, MgB_2_ benefits from a simple phase formation, making porosity control and pinning engineering the primary challenges. Amorphous B with fine Mg powder outperforms crystalline B or coarse Mg, and MgB_4_ precursor reduces shrinkage. Hydrocarbon dopants (580–660 °C) offer the strongest high-field enhancement, while metallic/oxide dopants favor low-field properties. The trade-off between doping uniformity and connectivity remains central. Future research should establish quantitative processing–structure–performance correlations to guide industrial fabrication. Notably, the *J*_c_ values of the carbon-doping routes discussed above were mostly obtained at 4.2 K and 10 T (the malic-acid-doped tape measured at 5 K and 8 T being the only exception), ensuring that the comparison reflects the intrinsic merits of each doping strategy rather than differences in measurement conditions.

## 5. Iron-Based Superconductors

### 5.1. Main Systems of Iron-Based Superconductors

The discovery of superconductivity at 26 K in F-doped LaFeAsO_1−x_F_x_ reported by Hosono’s group in 2008 pioneered the family of IBS, a class of layered high-temperature superconductors complementary to cuprate systems [95]. To date, the IBS family comprises numerous layered compounds, which are conventionally categorized into several prototype series according to the crystal symmetry of their undoped parent phases, with structural complexity increasing progressively.

The “11” family, typified by FeSe(Te), adopts the simplest tetragonal α-PbO-type structure, wherein the conducting FeSe layers are stacked directly without intervening charge reservoir layers [96,97]. Its intrinsic *T*_c_ is relatively low (~8 K), though it can be elevated through chemical doping or the application of external pressure. Owing to its structural simplicity, FeSe has long been regarded as a model system for investigating the high-*T*_c_ mechanism in IBS.

The “111” family, with representative members LiFeAs and NaFeAs, features double layers of alkali metal atoms that separate the conducting FeAs layers [98,99]. This family crystallizes in the PbFCl-type structure (space group P4/nmm) and exhibits a maximum *T*_c_ of approximately 18 K. However, the poor chemical stability of these compounds has limited their research and application potential.

The “122” family comprises parent compounds with a 1:2:2 atomic ratio, for example, A_E_Fe_2_Pn_2_ (A = Ba, Sr, K, Ca, Eu) [100,101]. Representative compositions are Ba_1−x_K_x_Fe_2_As_2_ and Sr_1−x_K_x_Fe_2_As_2_. This family crystallizes in the tetragonal ThCr_2_Si_2_-type structure (space group I4/mmm), with alkaline earth metal layers serving as charge reservoirs. Superconductivity is induced by hole doping (e.g., via K substitution) or electron doping (e.g., via Co substitution) into the FeAs layers. The maximum *T*_c_ of the 122 family surpasses 38 K. In particular, this system is characterized by low superconducting anisotropy, an exceptionally high upper critical field (Hc_2_), substantial current-carrying capacity, and remarkably slow degradation of transport properties under high magnetic fields.

The “1111” family, with a 1:1:1:1 stoichiometry, mainly includes LnOFePn (Ln = La, Ce, Pr, Y and other lanthanides; Pn = P, As) and AFeAsF (A = Ca, Sr and other alkaline earth metals). Rare earth and oxygen atoms form charge reservoir layers at an atomic ratio of 1:1. It adopts the tetragonal ZrCuSiAs structure with the space group P4/nmm, and belongs to the electron-doped system (though hole doping via specific substitutions is also achievable). The carriers are introduced mainly by partial substitution of oxygen with fluorine (F doping), which injects electrons into the FeAs conducting layers to achieve high *T*_c_. The 1111 family holds the highest *T*_c_ among all IBS, with a maximum value exceeding 50 K [102].

Novel structural families have also been discovered by designing layered compounds containing FeAs structural units. Notable examples include the “32225” family represented by Sr_3_Sc_2_O_5_Fe_2_As_2_ and the “42226” family represented by Sr_4_V_2_O_6_Fe_2_As_2_ [103,104,105].

**Figure 9:**
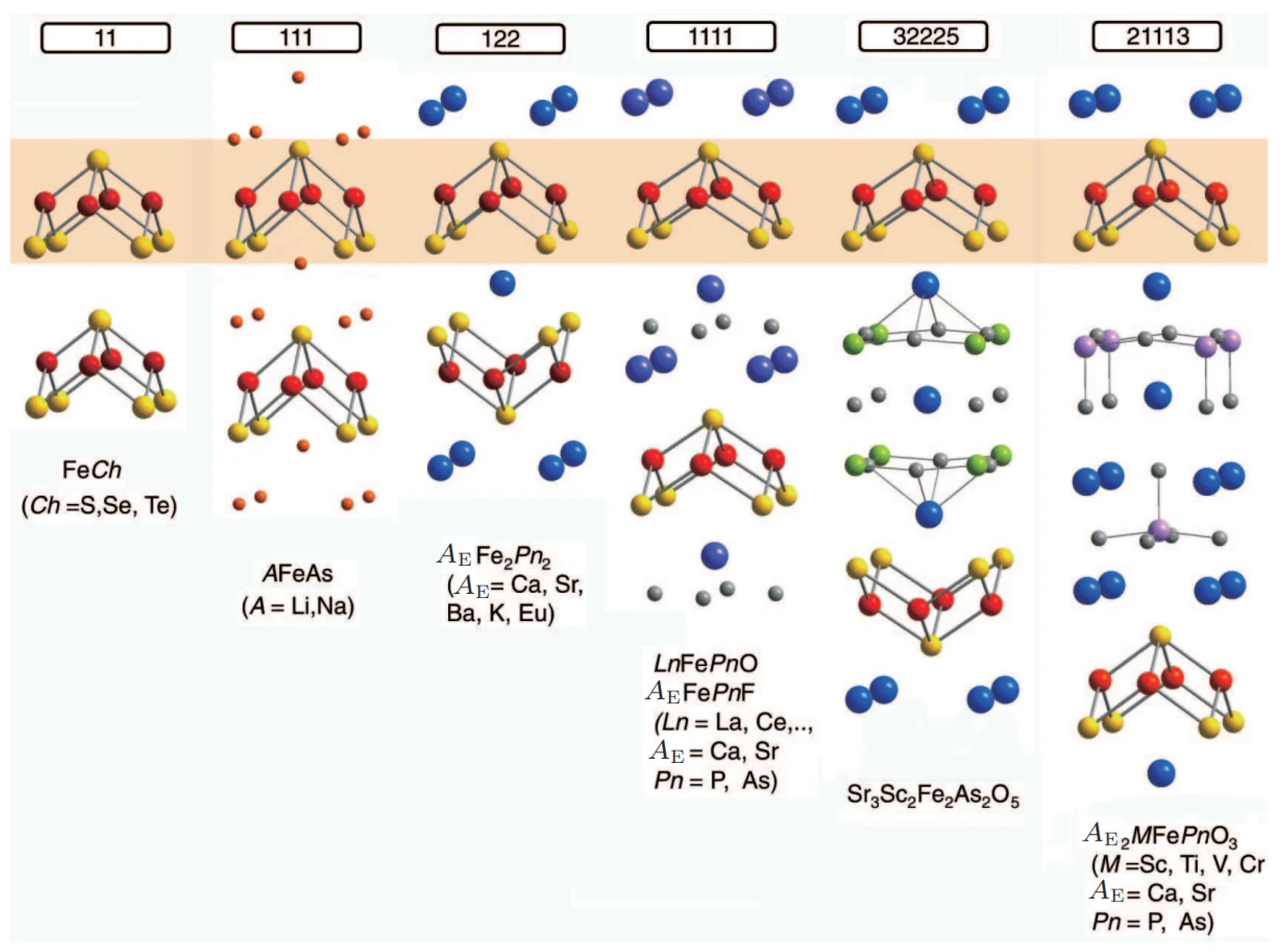
The crystal structures of Fe-based superconductors with 11-, 111-, 122-, 1111-, 21113-, and 32225-type structures. The FeCh/FePn (Pn: pnictogen, Ch: chalcogen) tetragonal blocks are highlighted in these structural models [106].

Among the four major IBS families, in the order of 11 → 111 → 122 → 1111, the two-dimensional character progressively strengthens while the three-dimensional character weakens, accompanied by a gradual increase in *T*_c_. The crystal structures of these families are shown in Figure 9.

**Figure 10:**
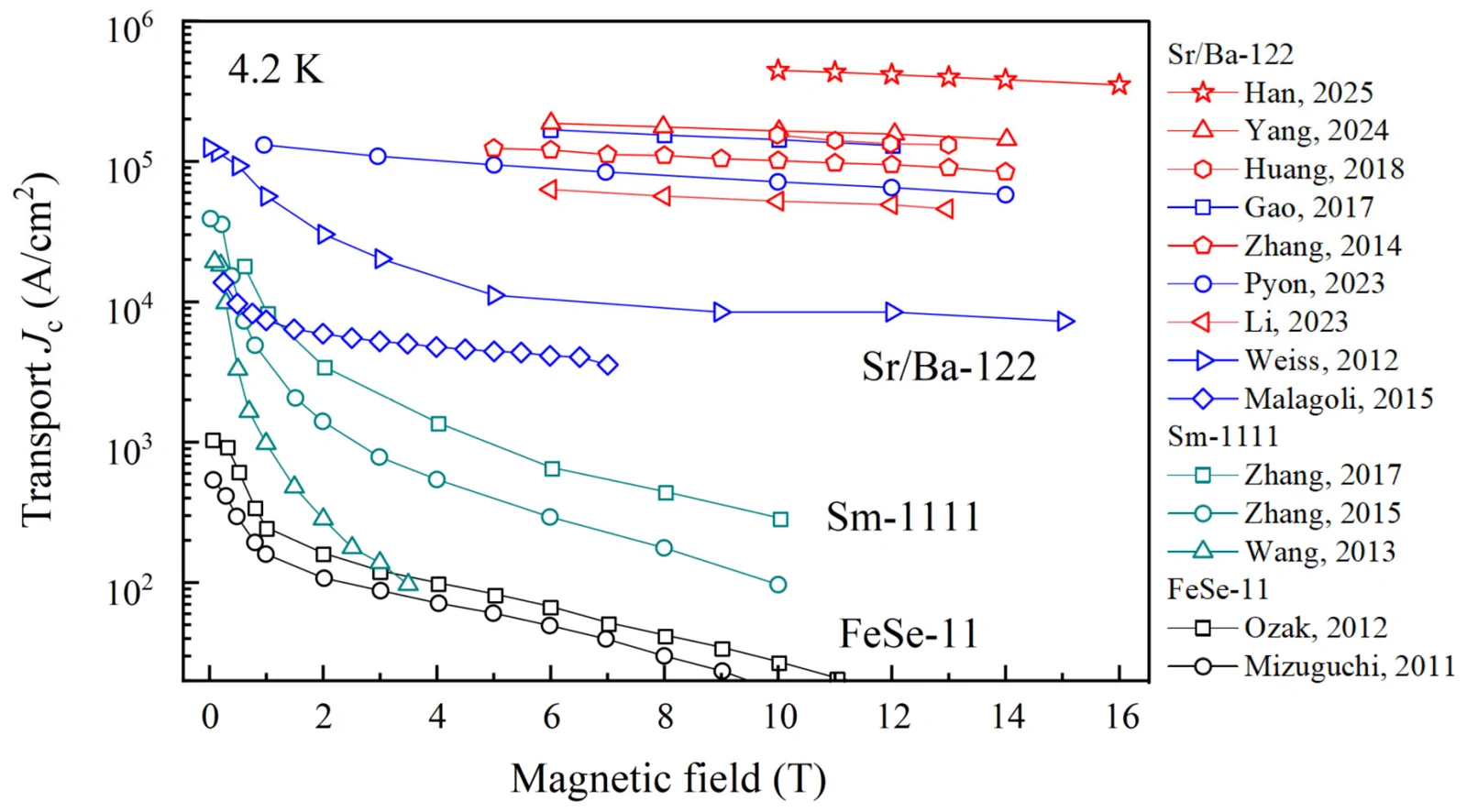
Transport *J*_c_ at 4.2 K as a function of applied magnetic field for PIT-processed 122, 1111, and 11 wires and tapes. The data refer to 11 wires [107,108], 1111 tapes [109,110,111], and Sr/Ba-122 wires and tapes [112,113,114,115,116,117,118,119,120].

From a practical application perspective, the 11 family suffers from a low critical temperature, which limits its practicality. The 111 family exhibits poor stability, resulting in limited research interest. The 1111 family possesses the highest *T*_c_ and upper critical field but suffers from strong anisotropy and difficulties in phase-pure synthesis due to fluorine volatilization. Although the *T*_c_ of the 122 family is lower than that of the 1111 family, it also delivers a high upper critical field and large critical current density. More importantly, the 122 family exhibits extremely low anisotropy, which is substantially lower than that of the 1111-type compounds. The development of IBS wires and tapes is illustrated in Figure 10. In summary, the 122 family presents the best overall performance among IBS and holds the most promising application prospects. This paper, therefore, primarily reviews the precursor powders of Ba-122.

To place these IBS data in a broader context, it is instructive to compare the transport performance of Ba-122 wires with that of BSCCO and MgB_2_ conductors under their respective optimal operating conditions. At 4.2 K, Bi-2212 round wires deliver the highest current-carrying capability in very high fields, with engineering current densities exceeding 200 A·mm^−2^ at 45 T [15]; wires made from the optimized 521 precursor reach *J*_c_ values of approximately 7.6 × 10^5^ A·cm^−2^ at self-field [28,29] and 4.67 × 10^5^ A·cm^−2^ at 12 T [37], whereas Bi-2223 tapes provide a practical Je of about 10^4^ A·cm^−2^ at 77 K [17,18,19,20,21,22]. For MgB_2_, the strongest performance is obtained at 4.2–20 K and moderate fields: doped tapes achieve *J*_c_ values of 1.85 × 10^4^ A·cm^−2^ at 4.2 K and 10 T [87] and 3.8 × 10^4^ A·cm^−2^ at 10 T [88], and MgB4-precursor samples reach 7.8 × 10^4^ A·cm^−2^ at 20 K and 2 T [70]. For Ba-122, the data in Figure 10, including *J*_c_ values of 2.3 × 10^5^ A·cm^−2^ at self-field and 1.6 × 10^4^ A·cm^−2^ at 10 T [121], demonstrate competitive transport performance with excellent stability up to 27 T [118]. In summary, under optimal operating conditions, Bi-2212 remains the benchmark for ultra-high-field magnets at 4.2 K, MgB_2_ is the most cost-effective choice at 20–30 K and moderate fields, and Ba-122 offers an attractive balance of moderate *J*_c_ and high-field stability at 4.2 K with inexpensive raw elements.

### 5.2. Precursor Powders of Ba-122 Iron-Based Superconductors

Early attempts to fabricate Ba-122 wires employed the in-situ powder-in-tube (PIT) method, wherein elemental powders of Ba, K, Fe, and As were directly loaded into metal tubes, and the superconducting phase was formed in-situ by heat treatment after mechanical deformation. However, this approach suffers from fundamental drawbacks: (1) the high reactivity of the four elements makes it difficult to completely avoid oxidation even under inert atmospheres during powder mixing and tube loading; (2) volatilization of K and As during heat treatment causes compositional deviation and precipitates non-superconducting impurity phases such as FeAs and elemental Fe; and (3) volume changes accompanying the in-situ reaction lead to low density of the final wires. Owing to these issues, the *J*_c_ of wires fabricated by the in-situ PIT method has long remained below 10^4^ A·cm^−2^.

In contrast, the ex-situ PIT method completes the phase formation reaction in advance. High-purity Ba-122 main phase is already formed in the precursor powders, and subsequent heat treatment only serves for sintering and densification without complex phase transformation, which remarkably improves wire performance [122,123]. Accordingly, the ex-situ PIT method has become the mainstream technology for manufacturing long Ba-122 wires. High-quality Ba-122 precursor powders for engineering production require high main-phase purity, high density, and excellent compositional uniformity.

At present, solid-state reaction sintering is the dominant route for preparing Ba-122 precursor powders. The detailed process is as follows: high-purity raw materials are thoroughly mixed in a planetary ball mill to ensure uniform composition. The mixed powders are then pressed into pellets, sealed in metal tubes, and sintered at high temperature under continuous argon protection to obtain the target precursor powders. Raw material purity, elemental ratio, and heat treatment parameters are the key factors determining the quality of final precursors, and a series of systematic optimization studies have been carried out worldwide.

Elemental Ba, K, Fe, and As are prone to oxidation and hydrolysis when exposed to oxygen and moisture in air. Impurities introduced by raw materials will lead to massive precipitation of non-superconducting impurity phases during high-temperature sintering. For this reason, the entire fabrication process of Ba-122 precursor powders, including weighing, ball milling, pellet pressing, sealing, and sintering, must be conducted in an argon-filled glovebox. Apart from adopting high-purity raw materials to reduce impurity introduction, researchers have developed multiple techniques to inhibit oxidation.

Dong et al. proposed a two-step solid-state synthesis route for Ba-122 precursors. The conventional one-step method directly mixes and sinters Ba, K, Fe, and As according to stoichiometric ratios, where highly reactive alkali metals are easily oxidized. By contrast, the two-step method firstly synthesizes intermediate phases KAs and BaAs separately, and then mixes them with the remaining raw materials for further sintering. This route mitigates raw material oxidation and effectively increases the purity of the superconducting main phase [9].

To mitigate the reactivity of the starting materials, Zaikina et al. substituted conventional elemental Ba and K with BaH_2_ and KH, respectively, and successfully synthesized the Ba-122 precursor. The resulting sample exhibited a *T*_c_ of 38 K, consistent with that of precursors prepared by the conventional route [124].

In a related study, Huang et al. employed KF as an alternative potassium source instead of elemental K, and the obtained Ba-122 bulk precursors showed comparable *T*_c_ and superconducting performance to those synthesized using K [125]. These findings offer new perspectives for reducing the chemical activity of Ba-122 precursor powders.

The preparation of Ba-122 precursors includes high-energy mechanical ball milling and high-temperature sintering. Since K has a low boiling point, significant volatilization loss occurs during these two stages, resulting in K deficiency in the system and further inducing various impurity phases. Therefore, excess potassium source is generally added to compensate for K volatilization in practical production.

Studies by Wang et al. indicate that appropriate excess K doping enhances the magnetic *J*_c_ of Ba-122 tapes without damaging the crystal structure or lowering the *T*_c_ [126]. Cheng et al. quantitatively investigated the correlation between K doping level and *J*_c_ in (Ba_1−x_K_x_Fe_2_As_2_) PIT tapes. A divergent doping dependence was observed for intra- and intergranular *(J*_c_): the intragranular (*J*_c_) maximizes at x ≈ 0.287 (underdoped region), while the optimal intergranular (*J*_c_) emerges at slightly overdoped (x ≈ 0.458), exceeding the benchmark for practical superconducting applications. The grain boundary transparency factor (ε = *J*_c_^inter^/*J*_c_^intra^) increases monotonically with K content, an effect that TEM-EDS analysis attributes to the formation of pervasive FeAs wetting layers at grain boundaries, which constitute SNS Josephson junction networks mediating intergranular supercurrent transport. Overdoping elevates carrier density and metallicity within superconducting grains, strengthening the proximity effect and further enhancing intergranular supercurrent. The composition (x ≈ 0.458) was recommended for practical wire fabrication due to its balanced superconducting performance. Crucially, the enhanced intergranular transport originates from amplified proximity effect in SNS junctions, rather than lattice distortion or grain growth, given the doping-independent grain size revealed by statistical microstructural analysis. In addition to K, the stoichiometric ratio of As also affects the final phase purity of precursors [127]. Tu et al. investigated residual Fe particle impurities in precursors and found that As deficiency is the primary cause. Adding excess As can eliminate Fe impurities and improve the transport performance of final superconducting tapes [128].

**Figure 11:**
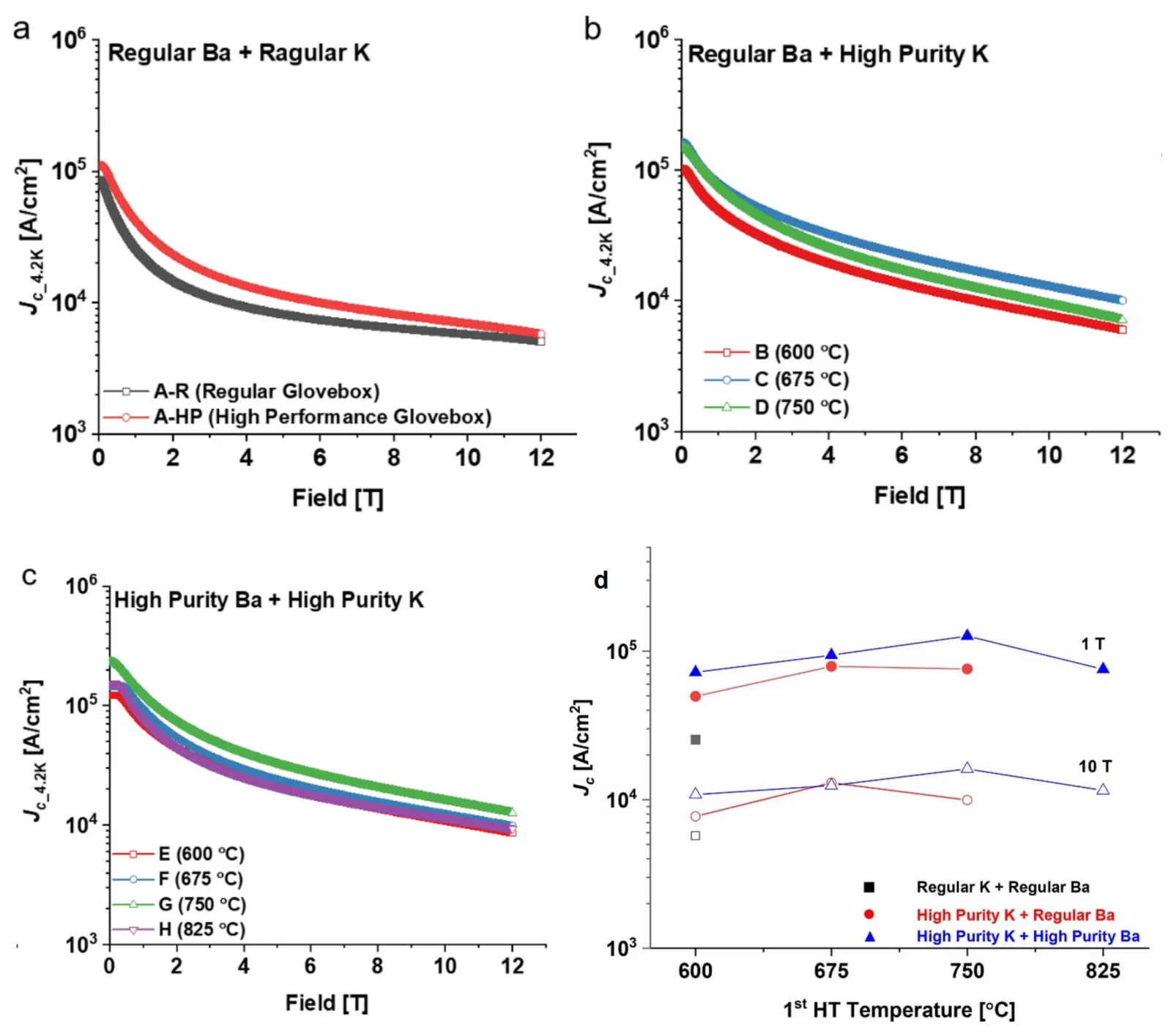
(**a**–**c**) *J*_c_ (H) plots for Ba_0.6_K_0.4_Fe_2_As_2_ bulks prepared under different conditions; (**d**) Effect of high-purity starting materials on *J*_c_ measured at 4.2 K (solid points: 1 T, hollow points: 10 T). The data in (**a**–**d**) are adapted/redrawn from Figures 3 and 7 of Pak et al. [121], respectively, to highlight the optimal sintering temperature at 750 °C (Sample G).

Thermal process regulation is an important approach to suppress impurity phase precipitation and optimize the microstructure of precursor powders. To optimize the sintering process, Pak et al. systematically varied the first heat-treatment (HT) temperature of K-doped Ba-122 precursors. As summarized in our Figure 11 (with data adapted from their Figures 3 and 7), the optimal sintering temperature was identified as 750 °C. The sample processed at this temperature (Sample G) exhibited the best current-carrying performance, achieving a *J*_c_ of 2.3 × 10^5^ A·cm^−2^ at self-field and 1.6 × 10^4^ A·cm^−2^ at 10 T [121]. Weiss et al. adopted a two-step sintering process to precisely control the reaction rate across different temperature ranges, which effectively suppresses the formation of non-superconducting impurities such as FeAs, enhances the phase purity of Ba-122, and thereby improves the *J*_c_ of the resulting bulks [129].

To synthesize Ba-122 precursors with high purity and uniformity, Malagoli et al. carried out heat treatment using three types of furnaces: a stationary furnace, an oscillating furnace, and a self-developed rotating furnace. The results show that continuous rotation in the custom furnace enables thorough reaction of well-mixed precursors and reduces FeAs and Fe impurities [130]. Furthermore, advanced densification technologies including hot pressing (HP), hot isostatic pressing (HIP), and spark plasma sintering (SPS) can optimize the microstructure, reduce internal pores and defects, and further enhance the overall performance of IBS precursors [131,132,133].

For Ba-122, the link between powder quality and conductor performance is governed by two quantities that are less routinely specified than for other superconductor systems: the absolute density of the reacted core and the K–As stoichiometric balance. Density matters because transport in Ba-122 is percolative through a granular network with limited grain-boundary transparency; a porous core therefore reduces *J*_c_ roughly in proportion to the loss of effective cross-section, and much of the improvement obtained by hot isostatic pressing after PIT deformation arises from densification [132]. The stoichiometric balance matters because the relevant deviation is not a simple potassium deficiency but a K–As imbalance: potassium loss during milling and sintering shifts the starting charge away from the target stoichiometry and can promote secondary phases, whereas excess K added to compensate for volatilization consumes As and leaves unreacted Fe particles that locally suppress superconductivity and obstruct current flow [128]. Separately, FeAs-type segregation at grain boundaries can form superconductor–normal–superconductor (SNS) junctions, whose transparency depends on the normal-layer thickness and the proximity effect; overdoping can strengthen this proximity coupling and thereby enhance intergrain supercurrent [127]. Because such stoichiometric deviations develop during milling and sintering and are difficult to detect by XRD at low impurity fractions, process specifications must rely on the starting charge composition and mass loss upon sintering rather than phase analysis alone. The recent achievement of high engineering critical current density in 122-type conductors [134] underscores that conductor-level performance requires simultaneous control of core densification and starting stoichiometry, together with suppression of detrimental impurity phases.

Taken together, Ba-122 precursor quality depends on three key factors: raw-material protection, stoichiometric compensation, and thermal treatment. Full argon atmosphere with two-step sintering avoids oxidation; excess K and As compensate volatilization, suppress FeAs/Fe impurities, and create pinning centers; optimized segmented sintering and advanced densification prevent decomposition and abnormal grain growth. Synergistic control yields high-purity, dense, and uniform precursors for ex-situ PIT processing. Unlike Bi-based phase engineering and MgB_2_ porosity control, Ba-122 prioritizes compositional accuracy: minor K/As deviations generate harmful FeAs/Fe phases that degrade connectivity. Accordingly, the ex-situ PIT route, using pre-synthesized superconducting powder, is more practical than the in-situ route for long-length Ba-122 wires.

## 6. Conclusions and Outlook

### 6.1. Conclusions

The PIT route is central for long superconducting wires, with precursor powders governing filling, deformation, reaction, densification, and *J*_c_. High-quality powders demand precise stoichiometry, controllable phase, tailored particles, low impurities, sheath compatibility, and batch consistency. Practical applications require longitudinal uniformity and scalability beyond lab-scale peak performance. Dominant bottlenecks are system-specific:

Bi-based: phase competition and kinetic control (stoichiometry, O_2_, carbon);

MgB_2_: Mg loss and ~23.3% shrinkage cause porosity; use low-oxygen Mg, amorphous B, MgB_4_, and balanced doping;

Ba-122: oxidation/volatilization of Ba, K, As require inert protection, excess K/As, and optimized sintering; ex-situ PIT is preferred.

Common challenges include impurity suppression, homogeneity, batch stability, heat-treatment matching, and long-length uniformity. Table 3 summarizes these key differences and commonalities across the three systems. Cross-system differences stem from thermodynamics and elemental behavior, yet all require precursor design integrated with deformation and heat treatment. Future work should shift from isolated powder optimization to an integrated framework linking composition, phase, microstructure, processing, and final performance. To further strengthen the comparison, Table 4 summarizes the main precursor preparation methods and their advantages and limitations for the three systems.

### 6.2. Challenges of Impurity Control, Densification, and Long-Length Conductor Uniformity

Impurity control, densification and long-length uniformity are the three most critical engineering challenges that bridge precursor development and practical conductor manufacturing. (i) Impurity control: for Bi-based conductors, residual carbon and secondary phases (e.g., Bi-2201 and 14:24 AEC) in the precursor are directly inherited by the filaments and degrade intergranular connectivity; for MgB_2_, oxygen uptake of the Mg and B powders and the formation of MgO at grain boundaries remain the dominant impurity issue, while chlorine contamination in certain boron grades was found to reduce compactness and *J*_c_; for Ba-122, oxide impurities formed by Ba and K upon exposure to O_2_/H_2_O at grain boundaries block intergranular current and can be suppressed only by inert-atmosphere handling and high-purity raw materials. (ii) Densification: the intrinsic porosity of in-situ PIT MgB_2_ cores (≈23.3% volume shrinkage) and the residual voids of Bi-2212 partial-melt processing limit the effective current-carrying area; mechanical milling, internal Mg diffusion, and hot isostatic pressing (HIP) or hot pressing have been proven effective densification routes for all three systems. (iii) Long-length uniformity remains challenging because powder-filling fluctuations, Mg/B particle-size mismatch, and carbon agglomeration can cause microstructural non-uniformity and local inhomogeneity after heat treatment, potentially scattering *J*_c_. Nevertheless, a 2.1 km-class carbon-doped MgB_2_ wire fabricated by optimized 3D vibrating powder filling exhibited only ≈2% *J*_c_ variation along its length at 20 K/2 T; the strong *J*_c_–n proportionality establishes the n-value as a practical industrial quality-control metric for uniform long-length conductors [155].

### 6.3. Outlook

To accommodate the escalating requirements of practical superconducting applications, future research on high-performance PIT precursor powders should prioritize the following targeted research directions:

(1) Establishment of a standardized powder evaluation system. Current laboratory investigations predominantly rely on X-ray diffraction phase identification and short-sample *J*_c_ characterization, while critical engineering-oriented indices, including particle size distribution, residual oxygen/carbon/hydrogen contents, elemental compositional uniformity, tap density, and powder flowability, are largely underemphasized. A unified and standardized evaluation framework is needed to quantitatively correlate intrinsic powder characteristics with macroscopic wire performance metrics, including *I*_c_, *J*_c_, n-value, core porosity, and long-length structural and performance uniformity.

(2) Synergistic optimization of precursor design and PIT processing protocols. The optimal precursor state is defined not only by superior powder-level properties, but also by excellent compatibility with subsequent wire drawing, rolling, and thermal treatment procedures. For each superconducting system, a closed-loop optimization mechanism spanning “precursor structural parameters-mechanical processing behavior-phase evolution kinetics-final superconducting performance” should be established to realize process compatibility and integrated optimization.

(3) Exploration of innovative precursor architectures and composite sheath matching strategies. Novel structural design avenues offer potential to overcome existing performance limitations. For instance, core–shell structured Mg powder can effectively suppress Mg volatilization in MgB_2_ systems; artificial pinning centers can be pre-incorporated into Bi-2212 precursor powders to enhance flux pinning capability; in addition, the interfacial reaction mechanisms corresponding to different sheath materials (e.g., pure Ag, Cu/Nb composite) in Ba-122 wires require systematic investigation to broaden the material and structural design window of PIT conductors.

## Figures and Tables

**Table 1:** Quantitative comparison of production cost and scalability metrics for PIT-processed Bi-based, MgB_2_, and Ba-122 conductors.

Comparison Item	Bi-Based (Bi-2212/Bi-2223)	MgB_2_	Ba-122 IBS
Representative sheath material	Ag (only chemically compatible, oxygen-permeable sheath)	Fe, Cu, or Nb (low-cost)	Cu/Ag or Ag (moderate cost)
Typical heat-treatment window	Approximately 830–890 °C (partial melting)	580–900 °C (doped systems)	Approximately 850 °C (two-step sintering)
Atmosphere requirement	Flowing O_2_	Inert or low-oxygen atmosphere	Full argon glovebox handling
Industrial-scale powder synthesis	Spray pyrolysis; kg-scale co-precipitation	Solid-state mixing and ball milling	Two-step solid-state reaction (ex-situ)
Main scalability bottleneck	Ag cost; narrow phase-stability window	Porosity control; doping uniformity; long-length uniformity	Strict inert protection; K/As volatilization compensation

**Table 2:** Summary of key processing-control factors and their corresponding effects on the microstructure and superconducting properties of conductors.

Material Characteristic	Key Controlling Factor	Effect on Final Properties
Chemical homogeneity	Reaction uniformity	Main phase purity, *J*_c_
Phase composition controllability	Phase formation pathway	Phase purity, texture, grain connectivity
Particle morphology and size	Packing density and densification	Porosity, *J*_c_
Sheath-material compatibility	Interfacial reaction	Long-length stability, thermal robustness

**Table 3:** Comparison of key characteristics of precursor powders for Bi-based, MgB_2_, and Ba-122 superconductors.

Comparison Item	Bi-Based (Bi-2212/Bi-2223)	MgB_2_	Ba-122 IBS
Primary bottleneck	Multi-phase competition; narrow phase stability region	Mg volatilization and volume shrinkage; high porosity	Oxidation/volatilization of reactive elements; compositional deviation
Key control parameters	Stoichiometry (Sr/Ca, Bi/Cu), Pb doping, carbon residues, oxygen partial pressure	Mg/B particle size, oxygen content, boron morphology, dopant dispersion	K/As compensation, inert protection, intermediate phases, sintering temperature
Main preparation routes	Spray pyrolysis/co-precipitation; two-powder route for Bi-2223	Solid-state mixing + mechanical alloying; chemical doping	Two-step solid-state reaction; ex-situ PIT using pre-synthesized superconducting phase
Performance influence mechanism	Secondary phases cause weak links; nano-Bi-2201 acts as pinning centers; Pb modulates kinetics	Pores reduce effective area; doping introduces lattice distortion and pinning; secondary phases improve connectivity	FeAs/Fe impurities block intergranular current; K content affects proximity effect; density determines transport
Engineering challenges	Compositional uniformity, carbon residue control, batch stability, narrow heat-treatment window	Mg oxidation, boron agglomeration, scale-up of doping, long-wire densification	Strict oxygen/moisture exclusion, precise K/As compensation, phase purity maintenance
Common challenges	Impurity suppression, compositional homogeneity, batch consistency, heat-treatment window matching, long-length uniformity		

**Table 4:** Comparison of the main precursor preparation methods for Bi-based, MgB2, and Ba-122 superconductors: advantages and limitations.

System	Synthesis Route	Process Characteristics	Advantages	Limitations
BSCCO Bi-2212, 85–92 K Bi-2223, 110 K	Solid-state reaction [55,135,136,137]	Stoichiometric oxides and carbonates (Bi_2_O_3_, PbO, SrCO_3_, CaCO_3_, CuO) are ball-milled, pelletised and calcined with intermediate crushing in air or under a controlled O_2_ partial pressure at 800–830 °C until the 2212/2223 assemblage forms.	Low-cost raw materials, simple equipment and good batch-to-batch reproducibility; the cation ratio is set directly, which facilitates doping and process tuning.	Homogeneity is limited to the micrometre scale, so unreacted oxides and secondary phases (alkaline-earth cuprates, CuO) persist; repeated long calcination implies high energy consumption and low throughput; the powder obtained is coarse and poorly reactive.
	Oxalate co-precipitation [7]	Bi/Sr/Ca/Cu (and Pb) nitrate solutions are precipitated with oxalic acid/ammonium oxalate at a controlled pH (≈2.9) and ageing time, then washed, dried and calcined in several steps.	Ionic-level mixing and precise cation stoichiometry; fine (<3 μm), high-surface-area and highly reactive powder; residual carbon can be tuned through the calcination schedule.	The precipitation windows of the individual cations differ, so sequential precipitation and segregation may occur; NH_4_^+^ and oxalate must be washed out; the flow is long and the batch size is limited (3 kg per batch demonstrated at pilot scale).
	Spray pyrolysis [8,138,139,140]	A mixed nitrate solution is atomised into microdroplets and pyrolysed in a hot zone, so that solvent evaporation, salt decomposition and oxidation are completed within seconds, yielding spherical, porous oxide particles.	One-step continuous synthesis with high throughput; mixing is uniform within each droplet, giving a narrow size distribution and a low carbon content (<80 ppm); the fine, reactive powder accelerates Bi-2223 formation and suppresses secondary phases; established for oxide-powder-in-tube (OPIT) precursor production.	High capital cost and strongly coupled process parameters; volatilisation of Pb and Bi requires compositional compensation; residual nitrate and closed pores may remain; the phase assemblage is sensitive to the calcination window.
MgB_2_ *T*_c_ = 39 K	In-situ PIT [56,141]	Mg and B powders (Mg:B ≈ 1:2) are blended and packed into a metal tube, and MgB_2_ forms in situ during the final heat treatment; nano-SiC, carbon sources or MgH_2_ are often added.	Low raw-material cost and simple processing; fresh Mg/B interfaces give better grain-boundary contact and connectivity than the ex-situ route; carbon or SiC addition introduces B-site substitution and additional pinning, raising *J*_c_ in high fields.	The reaction involves a ≈23% volume contraction and Mg volatilisation, leaving a porous, low-density core; the core is sensitive to Mg purity, oxygen and atmosphere (MgO formation); the heat-treatment window is narrow.
	Ex-situ PIT [142,143,144]	Phase-pure MgB_2_ powder is pre-synthesised (optionally doped, milled and classified) and then packed, deformed and sintered.	The phase composition is fixed before deformation, so there is no Mg volatilisation or abrupt volume change; good dimensional and process stability; the powder can be pre-modified (milling, chemical treatment); working and heat treatment are reproducible.	MgO and hydrated surface layers decouple the grains; the low reactivity hinders densification, so *J*_c_, especially in high fields, is generally lower than that of in-situ wires; *J*_c_ degrades on ageing.
	IMD (internal Mg diffusion) [145,146,147]	A central Mg rod is surrounded by B powder (often with carbon or SiC addition); during heat treatment liquid Mg diffuses radially into the B layer and a dense MgB_2_ layer grows outwards from the core.	The in-situ reaction yields a dense MgB_2_ layer with well-connected grains; *J*_c_ of the order of 10^5^ A cm^−2^ at 4.2 K and 10 T has been reported, far above that of conventional PIT wires, and Mg loss is low; the geometry is compatible with multifilamentary long lengths.	Assembly is complex and process control is demanding; the finite diffusion distance leaves unreacted B, and infiltration and layer thickness are non-uniform; the thick sheath and residual porosity restrict the engineering current density *J*_e_; cost is high.
Ba-122 Ba_1−x_K_x_Fe_2_As_2_ *T*_c_ ≈ 38 K	In-situ PIT [148,149,150,151]	Elemental Ba, K, Fe and As are mixed in a glove box, packed into a metal tube and reacted in situ during the heat treatment that follows deformation.	Few processing steps and a short flow; the sealed sheath suppresses K and As volatilisation; in-situ grain boundaries favour connectivity; wires and tapes are obtained directly by PIT.	K is highly reactive and hygroscopic, requiring anhydrous and oxygen-free handling; K loss causes compositional deviation; incomplete conversion leaves FeAs, residual Fe and porosity.
	One-step solid-state synthesis [152,153]	Ba, K, Fe and As (or selected binary compounds) are mixed in stoichiometric proportions, ball-milled, pelletised, sealed and sintered in a single high-temperature step.	Simplest flow and short cycle; no intermediate compounds are required; suitable for batch powder synthesis and for single crystals or bulk specimens.	K and As losses cannot be compensated within one step, which degrades compositional and phase reproducibility; BaO/K_2_O and FeAs impurities form readily; density and phase purity are difficult to achieve simultaneously.
	Two-step solid-state (KAs/BaAs) [151,154]	KAs and BaAs are synthesised and purified separately, then blended with Fe and sintered to form the 122 phase.	The intermediates can be pre-purified with controlled composition and particle size, and direct contact between K and As is avoided; the staged reaction proceeds more completely, with less secondary phase and grain-boundary segregation; *T*_c_ and *J*_c_ are more reproducible.	More processing steps and a longer cycle, with two rounds of weighing, milling, pressing and sealing; K volatilisation, oxidation and contamination risks persist; labour and energy demand are higher.

## Data Availability

No new data were created or analyzed in this study. Data sharing is not applicable to this article.

## Abbreviations

AEC, alkaline-earth cuprate; AIMI, advanced internal magnesium infiltration; BET, Brunauer–Emmett–Teller; BSCCO, Bi–Sr–Ca–Cu–O; CCO, Ca–Cu–O; CF, copper-free; CICC, cable-in-conduit conductor; CP, co-precipitation; CSD, chemical solution deposition; EDS, energy-dispersive X-ray spectroscopy; HIP, hot isostatic pressing; HP, hot pressing; IMD, internal magnesium diffusion; *J*_c_, critical current density; *J*_e_, engineering critical current density; MOCVD, metal–organic chemical vapor deposition; PIT, powder-in-tube; PLD, pulsed laser deposition; REBCO, rare-earth barium copper oxide; SP, spray pyrolysis; SPS, spark plasma sintering; XRD, X-ray diffraction.
